# The Effect of Pristine and Hydroxylated Oxide Surfaces on the Guaiacol HDO Process: A DFT Study

**DOI:** 10.1002/cphc.202100583

**Published:** 2021-09-30

**Authors:** Fabian Morteo‐Flores, Alberto Roldan

**Affiliations:** ^1^ Cardiff Catalysis Institute School of Chemistry Cardiff University Main Building, Park Place CF10 3AT Cardiff UK

**Keywords:** biomass valorisation, density functional calculations, hydrodeoxygenation, hydroxylated surfaces, surface chemistry

## Abstract

The acid‐base character of oxide supports is crucial for catalytic reactions. In this work, the acid‐base properties of five oxide surfaces common in heterogeneous catalysis were investigated and related to their interaction with monolignol compounds derived from lignin. We have used density functional theory simulations also to understand the role of the surfaces’ hydroxylation state. The results show that moderate hydroxyl coverage on the amphoteric γ‐Al_2_O_3_ (110) slightly strengthens the oxy‐compounds’ adsorption due to an increase in Lewis acidity. Similarly, low hydroxyl coverage on the reducible TiO_2_ (101) enlarges its adsorption capacity by up to 42 % compared with its clean surface. The higher affinity is attributed to the more favourable interaction between the surface‐OH groups and the aromatic rings. Overall, the results indicate that hydroxyl coverage enhances the amphoteric and reducible adsorption capacity towards aromatic species.

## Graphical abstract

**Hydroxylated or not**? The catalyst support is not always innocent during the catalytic hydrodeoxygenation (HDO) process, and its acid‐base character may define the catalytic performance. The present work uses accurate periodic density functional theory simulations to rationalise trends on the acid‐base properties of five clean and hydroxylated oxide surfaces, γ‐Al_2_O_3_, CeO_2_, MgO, β‐SiO_2_ and TiO_2_ anatase, and their affinity towards model compounds derived from the lignin pyrolysis process.

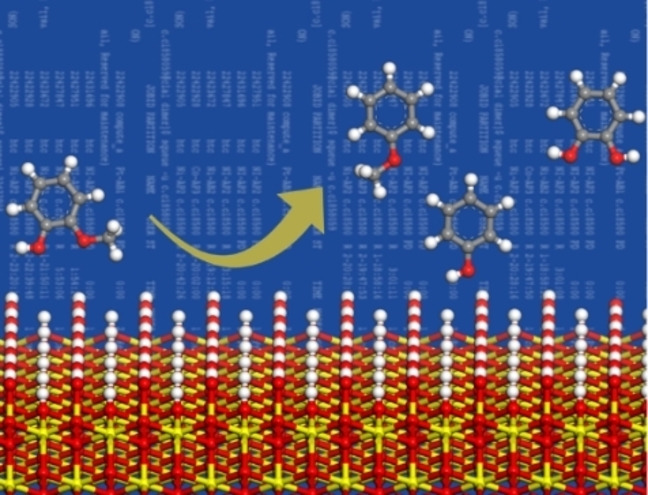

## 1 Introduction

**Figure 1:**
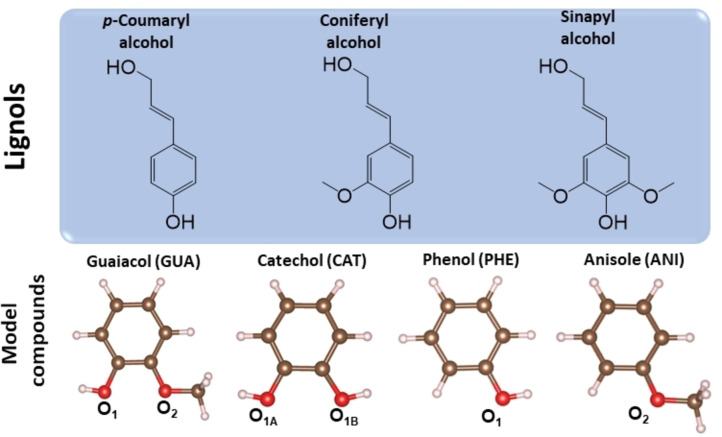
The three lignols building blocks of lignin and single model compounds resulting from the lignin fast‐pyrolysis.

The transformation of biomass into valuable bulk chemicals is crucial for a sustainable chemical industry and the implementation of a circular economy. Lignocellulosic biomass is the most promising form of biomass due to its abundance, non‐food nature and low cost. The three most significant components forming biomass are cellulose (33–51 %), hemicellulose (19–34 %) and lignin (20–30 %),^[1]^ being the latest the most thermally stable compound among them.^[2]^ Lignin has a complex structure mainly formed by three aromatic lignols – p‐coumaryl, coniferyl and sinapyl alcohol (Figure 1) – connected through a three‐dimensional network.^[3]^ As a result, the fast pyrolysis of lignin yields a mixture containing about 60 different oxygenated compounds.^[4]^ The high amount of oxygen present in such a mixture (28–40 wt.%) is responsible for its corrosiveness, low thermal stability and low energy density hindering its utilisation as biofuel.^[5]^ A common method to upgrade the biomass circularity is the hydrodeoxygenation (HDO) process, where the oxygens in the mixture are hydrogenated and removed as water.^[6]^

Due to the wide variety of lignin phenolic units, it is common to employ model compounds to understand and optimise the upgrading process. These model compounds should contain methoxy (−OCH_3_) and hydroxy (−OH) groups connected to an aromatic ring, representing the main functional groups in the pyrolysed mixture.^[7]^ Guaiacol (1‐hydroxy‐2‐methoxy benzene) has been commonly selected as a primary model because it contains these two main functional groups (see Figure 1).^[8]^ The principal products upon guaiacol HDO are:^[9]^ anisole,[^4^, ^8b^, ^10^] catechol,^[11]^ phenol,^[12]^ and benzene.^[11]^

Different catalytic materials have been studied to improve the HDO efficiency. Supported transition metals like Ni and Pt nanoparticles have received substantial attention due to their oxyphilic characteristics, helping to absorb oxygen compounds and easy desorption of deoxygenated products.^[13]^ These metal nanoparticles are commonly supported on oxides due to their easy preparation, stability, and accessible cost.^[14]^ There are two main aspects to be considered in the choice of supports for HDO catalysts: (i) negligible carbon precipitation, which is related to low surface acidity, and (ii) the ability to activate oxy‐compounds facilitating their reduction.^[15]^ Thus, to innovate and develop cost‐effective HDO catalysts, we must understand the supports’ chemistry under reaction conditions.^[16]^

Among common catalysts’ supports, γ‐Al_2_O_3_ is one of the most widely used in the HDO due to its excellent performance for activating phenolic compounds.[^12d^, ^17^] However, γ‐Al_2_O_3_ transforms into boehmite under hydrothermal conditions, leading to coke deposition.^[18]^ Alternatively, oxide supports such as MgO,^[19]^ CeO_2_,^[20]^ SiO_2_,[^8d^, ^21^] and TiO_2_ anatase [22] have been considered based on their acid‐base and reducibility properties. These oxide surfaces are hydroxylated in contact with water molecules under room conditions.^[23]^ Water interacts with the surface dangling bonds, and upon the transference of electrons, the water molecule dissociates forming surface‐OH groups.^**[**24]^ In this work, we examined the clean and hydroxylated oxides’ surfaces of γ‐Al_2_O_3_, CeO_2_, MgO, β‐SiO_2_ and TiO_2_ anatase and their interaction with model compounds derived from the lignin pyrolysis process.

## Computational Details

We have carried out spin‐polarised density functional theory (DFT) at 0 K using the Vienna Ab initio Simulation Package (VASP)^[25]^ to investigate the interaction of phenolic compounds with clean and hydroxylated oxides surfaces. The selected oxides surfaces were γ‐Al_2_O_3_, CeO_2_, MgO, β‐SiO_2_, and anatase‐TiO_2_ (a‐TiO_2_), and the compounds were guaiacol (GUA), anisole (ANI), catechol (CAT), phenol (PHE), and benzene (BEN). The exchange and correlation contributions were calculated using the generalised gradient approximation (GGA) with the revised functional of Perdew‐Burke‐Ernzerhof (RPBE).^[13]^ The core electrons were described using the Projected Augmented Wave (PAW) formalism.^[26]^ Dispersion interactions were added using the zero damping Grimme's dispersion correction DFT−D3.^[27]^ Dipole correction perpendicular to the surfaces of the oxides were corrected upon molecular adsorptions. The conjugate gradient convergence criteria were 0.03 eV Å^−1^ for the ionic and 10^−5^ eV for the electronic threshold. DFT+U method was applied for CeO_2_ oxide surface to describe the localisation of 4 f orbitals using the Liechtenstein method.^[28]^ Parameters for DFT+U method were set to 4 eV (U_eff_), which replicate the reduction of CeO_2_ with *J*=1 eV and *U*=5 eV.^[15c]^

The Digne model was chosen for simulating the γ‐Al_2_O_3_ structure.^[29]^ CeO_2_ bulk has a cubic fluorite structure (*Fm3 m* crystal structure), in which four Ce atoms are located in centre of a cubic lattice (Ce_7c_), and the O atoms occupy the tetrahedral lattice sites.^[30]^ MgO belongs to the group *Fm3 m* and contains one formula unit per primitive cell based on an Mg^2+^ with a neighbour O^2−^ atom.^[31]^ For SiO_2_, the β‐cristobalite structure was selected using a simple cubic formed with SiO_4_ tetrahedral units, i. e. a Si_4c_ at the centre and O_2c_ atoms at the corner ordered in the *Fd3 m* cubic structure.^[32]^ Bulk TiO_2_ unit cell for the anatase phase (a‐TiO_2_) contains four TiO_2_ units (12 atoms) where Ti atoms are in octahedral coordination with six O atoms, provoking inequivalent distances between Ti−O bonds in the structure (long and short bonds).^[33]^ The bulk structures are shown in Figure S1 in the Supporting Information. An optimised number of k‐points using the method of Monkhorst‐Pack was set to 13×13×13 k‐point grid, and a kinetic energy cut‐off of 550 eV was defined for the valence electron plane‐wave basis set (see Figure S2).^[34]^

Slab models of low index surfaces were generated with the METADISE code.^[35]^ We have chosen the (100), (110), (101), and (111) planes for the oxides’ surfaces. These slabs were built upon converge of surface energy (γ) as a function of slab thickness, vacuum, k‐points, and the number of atomic layers relaxed (see Figure S3). γ‐Al_2_O_3_ was modelled with a four‐layer slab, *p(2x2)* with 32 Al and 48 O atoms (surface area=81.2 Å^2^); for CeO_2_, we chose the oxygen‐terminated three‐layers of three atoms layers each, *p(4x4)* with 32 Ce and 64 O atoms (surface area=207.3 Å^2^). MgO was modelled with a four‐layer slab, *p(2x2)* of 32 Mg and 32 O atoms (surface area=70.9 Å^2^). For β‐SiO_2_, a four‐layer oxygen terminated slab, *p(4x4)* with 64 Si and 128 O atoms (surface area=223.2 Å^2^), was chosen. Finally, for a‐TiO_2,_ we selected a four‐layer oxygen terminated slab, *p(3x3)* with 48 Ti and 92 O atoms (surface area=118.9 Å^2^). The vacuum perpendicular to the surfaces is 15 Å, and the Brillouin zone was sampled with a Γ‐centred 3x3x1 k‐points grid. Isolated molecules were placed in a 20×20×20 Å^3^ box to avoid interactions with their periodic images.

## 2 Results and Discussion

### 2.1 Slab Calculation and Atomic Geometries

Table 1 shows the surface energy (γ) for each clean oxide surface calculated using Equation 1.

(1) $$\gamma =\frac{E_{slab}^{relax}-E_{bulk}}{A}-\frac{E_{slab}^{fix}-E_{bulk}}{2A}$$

**Table 1:** Calculated oxide surface energies in J m^−2^.

Termination *(hkl)*	γ‐Al_2_O_3_	CeO_2_	MgO	β‐SiO_2_	a‐TiO_2_
**(111)**	1.85	0.57	3.91	2.43	1.71
**(110)**	1.63	0.87	2.62	2.17	1.17
**(100)**	1.40	1.67	1.31	1.52	0.69
**(101)**	1.75	0.87	2.62	1.67	0.61

where $E_{slab}^{relax}$ is the total energy of one side relaxed slab, $E_{slab}^{fix}$ is the unrelaxed bulk‐terminated slab, E_bulk_ is the bulk energy, and A is the area of the generated surface.^[36]^

The surface energy trend for γ‐Al_2_O_3_ is (100)<(110)<(101)<(111) similar to previous reports.^[37]^ Although the (100) facet is the most stable, the (110) is commonly selected to represent the γ‐Al_2_O_3_ reactivity based on experimental studies confirming it to be the predominant surface, which coves ∼83 % of the total surface area.[^37b^, ^38^] The trends of surface energy for the other oxide surfaces are as follows: for CeO_2_ (111)<(110)=(101)<(100), for MgO (100)<(110)=(101)<(111), for β‐SiO_2_ (100)<(101)<(110)<(111) where O‐terminated slab is more stable than Si‐terminated slab, and for a‐TiO_2_ (101)<(100)<(110)<(111) Miller indices.^[39]^

**Figure 2:**
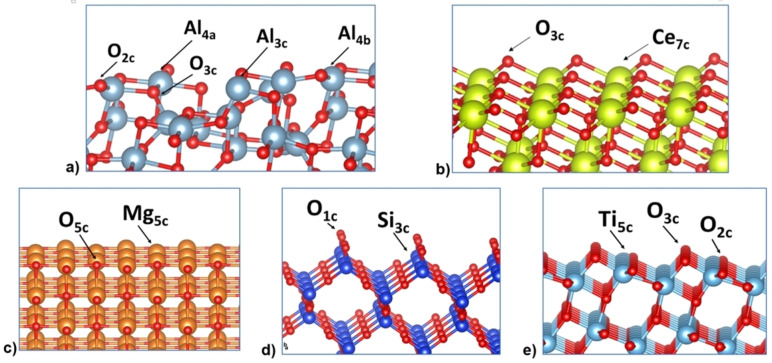
Side view of clean surfaces of a) γ‐Al_2_O_3_ (110), b) CeO_2_ (111), c) MgO (100) d) β‐SiO_2_ (100) cleaved bulk, and e) a‐TiO_2_ (101). O atoms are represented in red colour and Al, Ce, Mg, Si and Ti metals atoms are represented in blue, yellow, orange, dark blue and light blue respectively. Surface sites are labelled, including their coordination as a subscript.

γ‐Al_2_O_3_ (110) consists of three‐ and four‐fold coordinated Al atoms (Al_3c_ and Al_4c_) and two‐ and three‐fold coordinated O atoms (O_2c_ and O_3c_). It should be noted that the Al_3c_ site exists only on the (110) surface, and it is the most acidic site. The lower the Al atom coordination is, the stronger its Lewis acidity.[^37^, ^40^] The O‐terminated CeO_2_ (111) surface exposes three‐fold coordinated O atoms with seven‐fold coordinated Ce atoms (O_3c_, Ce_7c_, respectively).^[41]^ MgO (100) surface is a flat terrace exposing O and Mg atoms with five‐fold coordination each.[^31b^, ^42^] β‐SiO_2_ (100) cleaved bulk contains three‐fold Si coordination (Si_3c_) and one‐coordinated non‐bridging O atoms (O_1c_) at the topmost layer. Finally, a‐TiO_2_ (101) surface has a five‐fold coordination Ti atoms (Ti_5c_) and two‐ and three‐fold coordinated O atoms (O_2c_ and O_3c_). The representative slab surfaces for each oxide surface are shown in Figure 2.

### 2.2 Electronic Properties of Clean Surfaces

**Figure 3:**
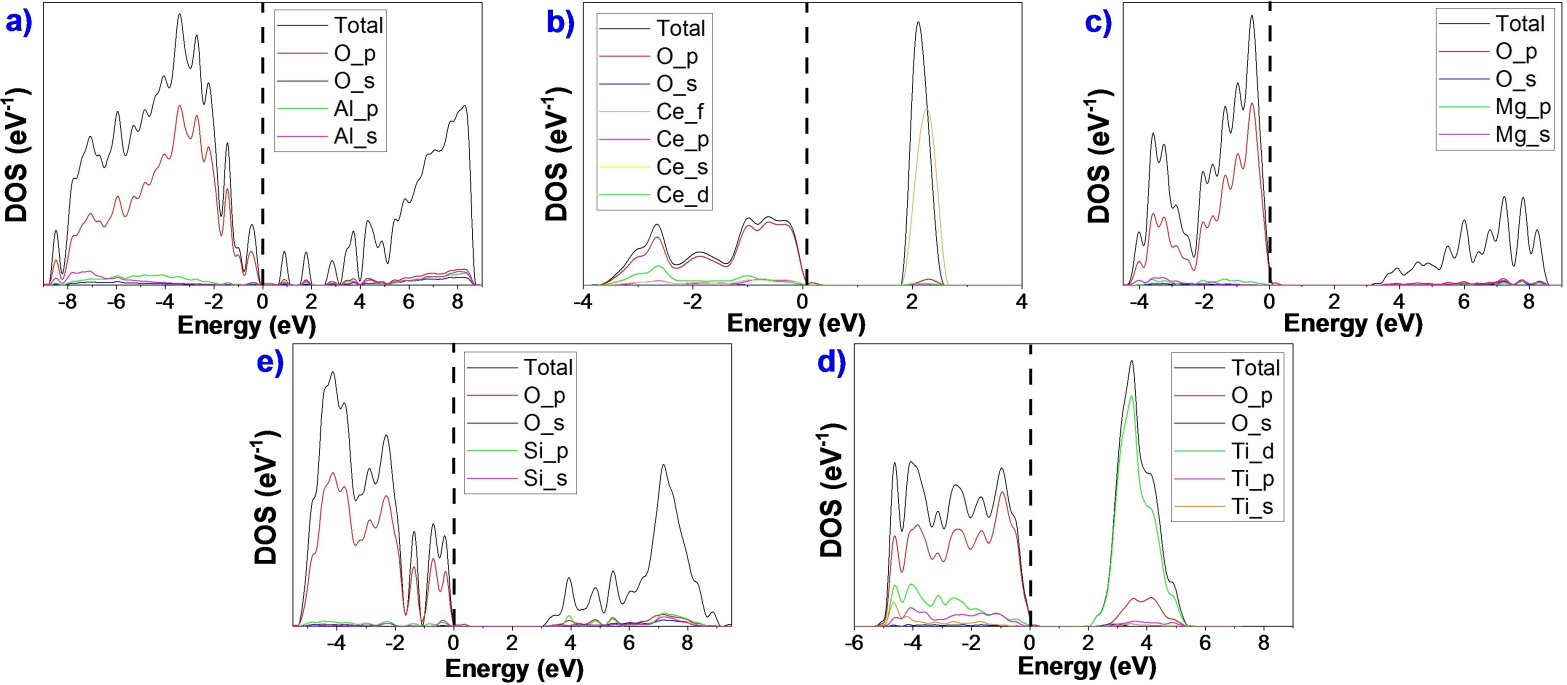
DOS and projected DOS for the most stable clean surfaces: a) γ‐Al_2_O_3_ (110), b) CeO_2_ (111), c) MgO (100), d) β‐SiO_2_ (100), and e) a‐TiO_2_ (101).

We employed the density of states (DOS) aligned to the Fermi energy to represent the electronic structure of the oxide surfaces (Figure 3 and Figure S4). All the DOS and projected density of states (PDOS) show two distinctive regions typical of an insulator: valence band (V_B_) and conduction band (C_B_). The valence bands (below 0 eV) of these oxides are mainly composed of O‐2p states, which slightly hybridise with the states of metals. The conduction band is formed by the unoccupied metal orbitals.

In γ‐Al_2_O_3_ (110), the O‐2p states dominate the valence band region and the C_B_ region is mainly composed of the O−Al antibonding orbitals, Figure 3a. The small bands between V_B_ and C_B_ correspond to the unoccupied surface dangling bonds. The resulting electronic structure is very similar to the one found by Yazdanmehr *et al*.^[43]^ The large degree of hybridisation on the γ‐Al_2_O_3_ oxide indicates a certain covalent character as previously identified.^[37a]^ In the O‐terminated CeO_2_ (111), the V_B_ region is composed by the hybridisation between O‐2p, Ce‐5d and 4 f states. The structure's main characteristic is the prominent peak in the C_B_ region formed by the localised empty Ce‐4 f states with a small contribution of O‐2p states indicating antibonding character.^[44]^ The PDOS shows a significant contribution of Ce to the V_B_ region, indicating a not completely ionic character.^[45]^ In the PDOS of MgO (100), the V_B_ region is predominated by O‐2p states with small contributions of Mg‐2p and 3 s states. Antibonding orbitals in the C_B_ region are composed mainly of Mg‐3 s states,^[46]^ showing that the material is primarily ionic.^[47]^ The β‐SiO_2_ (100)‐DOS has a V_B_ region formed by the Si‐3p and 3 s and O‐2p states. The dangling bonds of the surface split the O‐2p states creating two peaks close to the bandgap due to remain unbonded oxygen atoms (electron lone pairs).^[39d]^ The contributions of Si‐3p states in the occupied region compared to O‐2p states indicate that the material has a substantial covalent character. The DOS of a‐TiO_2_ (101) shows a strong hybridisation in the V_B_ region composed of O‐2p and Ti‐3d orbitals; this indicates a strong interaction between Ti and O with a band width of approximately 5.0 eV.^[48]^ In contrast, the C_B_ region comprises unfilled Ti‐3d states containing a significant contribution of O‐2p and 2 s states. This material presents a considerable covalent behaviour.^[49]^

The bulk and surfaces electronic structure main differences are related to the oxygen electron dangling bonds at the surface, which causes a decrease in the bandgap's size (see Table S2).[^31b^, ^50^] Although the DOS analysis gives information about the characteristics of the material, further research needs to be done to clarify the relationship between their acid/base properties and the compounds in HDO processes.

### 2.3 Hydrogen and Oxygen Adsorption

**Figure 4:**
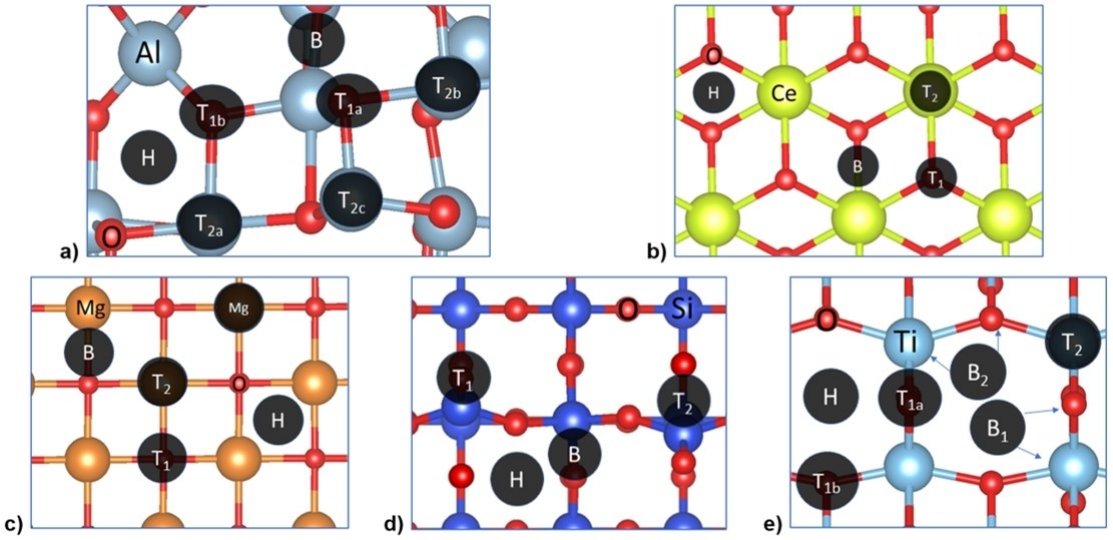
Top view of the a) γ‐Al_2_O_3_ (110), b) CeO_2_ (111), c) MgO (100), d) β‐SiO_2_ (100), and e) a‐TiO_2_ (110) slab models. The adsorption sites are indicated with a black circle labelled as hollow (H), bridge (B), and top (T) adsorption sites. Colour code: O atom is represented in red colour and Al, Ce, Mg, Si and Ti metals atoms are represented in blue, yellow, orange, dark blue and light blue, respectively.

We have investigated the O and H adsorptions on the oxides’ surfaces to identify their chemical groups’ affinity. Different adsorption sites have been considered, including top‐oxygen (T_1_), top‐metal (T_2_), bridge (B), and hollow (H), see Figure 4. For γ‐Al_2_O_3_ (110), we also found two inequivalent top‐oxygen sites: T_1a_ (O_2c_) and T_1b_ (O_3c_), and three top‐metal sites: T_2a_ (Al_4a_), T_2b_ (Al_4b_) and T_3c_ (Al_3c_). For a‐TiO_2_ (101), there are also two different top‐oxygen sites: T_1a_ (O_2c_) and T_1b_ (O_3c_), and two bridge sites: B_1_ (Ti‐O_2c_) and B_2_ (Ti‐O_3c_). The hydrogen and oxygen adsorption energies (E_ads_) were calculated using Equation (2); the E_ads/slab_ is the energy of the adsorbate on the slab, and E_adsorbate_ and E_slab_ are the energies of the free adsorbate (H_2_ and O_2_) and clean surface, respectively.

(2) $$E_{ads}=\;E_{ads/slab}-\left(E_{slab}+1/2{\;E}_{adsorbate}\right)$$

Table 2 contains only the information for the most stable adsorption sites on each surface. Bader charge analysis method was employed to measure the charge transfer between the surface and the adsorbed atoms. The compilation of adsorption energies, charge transfers and distances between the surface and the adsorbates are summarised in Table S3–S4.

**Table 2:** Calculated hydrogen and oxygen adsorption energies (E_ads_), Bader charge analysis (q) and distance between the atoms (H or O) – surface (d) for the most favourable adsorption sites.

		γ‐Al_2_O_3_ (110)	CeO_2_ (111)	MgO (111)	β‐SiO_2_ (100)	a‐TiO_2_ (101)
**H**	site	T_1a_ (O_2c_)	T_1_ (O)	T_1_ (O)	T_1_ (O)	T_1a_ (O_2c_)
	E_ads_ [eV]	−1.31	−1.15	−0.16	−1.00	−0.10
	q [|e^−^|]	0.70	0.60	0.63	0.66	0.07
	d [Å]	0.97	0.97	1.33	0.97	0.97
**O**	site	T_2c_ (Al_3c_)	H	T_1_ (O)	B (Si−O)	B_1_ (Ti−O_2c_)
	E_ads_ [eV]	−2.30	−1.44	−0.96	−0.48	−0.96
	q [|e^−^|]	−1.41	−0.52	−0.74	−0.75	−0.34
	d [Å]	1.76	1.32	1.54	1.64	1.44

In general, the most favourable adsorption site for H is on top of oxygen, leading to hydroxyl groups, while atomic oxygen prefers to adsorb on the metals.^[51]^ Overall, the O adsorption energy increases as it does the charge transfer from the surface to the O. On CeO_2_, the oxygen adsorption takes place competitively on the Ce‐atom and the hollow site, which can be helpful for molecules with two oxo groups such as catechol and guaiacol. The only exception found is on MgO, where O preferably adsorbs on top of surface‐O (T_1_ site) at 1.54 Å, similar to the bond length of a peroxo group (1.53 Å), indicating that the interaction results in a peroxide ion with the surface.^[52]^

According to Sabatier's principle, weak interaction between the surface's site and the oxygenated compounds, e. g. GUA, CAT, ANI, PHE, does not facilitate the removal of the O from the model compounds.^[53]^ Hence, based on our previous results, the HDO performance order should follow the oxygen adsorption energies, i. e. γ‐Al_2_O_3_>CeO_2_>a‐TiO_2_≈MgO>β‐SiO_2_. However, the accessibility of this site according to the surface morphology and steric hindrance should also be considered, see below.

### 2.4 Hydroxylated Surfaces

**Figure 5:**
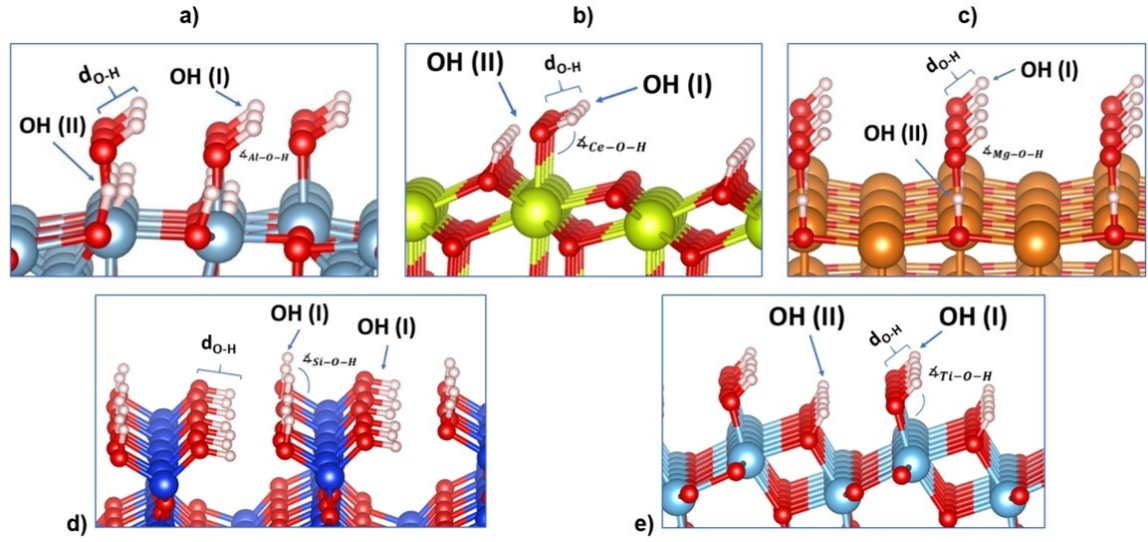
Schematic side views of the hydroxylated surfaces, where OH (I) corresponds to a terminal hydroxyl group and OH (II) a bridging hydroxyl group for a) γ‐Al_2_O_3_ (110), b) CeO_2_ (111), c) MgO (100), and e) a‐TiO_2_ (101). β‐SiO_2_ (100) (d) contains two geminal silanol OH (I). Colour code: O, H, Al, Ce, Mg, Si and Ti atoms are represented in red, white, blue, yellow, orange, dark blue and light blue colour, respectively.

The hydroxylation of oxide surfaces is achieved through the dissociation of water molecules on them. The OH is bonded to a cationic site, forming a terminal hydroxyl OH (I), whereas the hydrogen sticks to the surface oxygen, creating a bridging hydroxyl, OH (II), see Figure 5‐a, ‐b, ‐c, and ‐e.^[54]^ Instead of OH (II), the β‐SiO_2_ (100) surface contains two silanols on each Si (geminal silanol HO−Si−OH) with a bond length between 0.96 and 0.98 Å (Figure 5‐d).^[55]^

The concentration of hydroxyl groups on the surface (hydroxyl coverage) is determined by the temperature and the H_2_O pressure conditions. The hydroxyl coverage on γ‐Al_2_O_3_ (110) covers from 3.0 OH ⋅ nm^−2^ to 11.8 OH ⋅ nm^−2^ at a temperature between 500 and 1000 K.^[56]^ The lowest hydroxyl coverage (3.0 OH ⋅ nm^−2^) makes the surface highly acidic because of the unsaturated Al_3c_ site.[^37a^, ^57^] For CeO_2_ (111), the most stable structure for the (111) facet has a concentration of hydroxyl groups close to 4.0 OH ⋅ nm^−2^.^[58]^ MgO (100) hydroxylation in normal conditions is minimal due to its low hydrophilicity, i. e. water adsorption occurs at very low temperatures.^[59]^ The opposite is on β‐SiO_2_, which hydroxylates during its synthesis at around 4.0–4.9 OH ⋅ nm^−2^.^[60]^ The hydroxyl coverage on a‐TiO_2_ (101) may reach 7.0 OH ⋅ nm^−2^.^[61]^ Table 3 summarises the OH coverage investigated for the five surfaces and provides bond distances and angles registered between the surface and the OH groups.

**Table 3:** Calculated hydroxyl coverages, bond distances (d), and angle (∡) for the most stable hydroxylated surface configurations.

		γ‐Al_2_O_3_ (110)	CeO_2_ (111)	MgO (100)	β‐SiO_2_ (100)	a‐TiO_2_ (101)
**OH coverage [nm^−2^]**		6.15	3.85	5.64	3.59	5.05
**OH(I)**	d_M−O_ [Å]	1.82	2.26	1.87	1.67	1.88
	d_O−H_ [Å]	0.97	0.97	0.97	0.97	0.97
	∡_M−O−H_ [°]	113.4	126.4	127.4	112.7	119.8
**OH(II)**	d_M−O_ [Å]	1.91	2.37	2.11	1.63	2.04
	d_O−H_ [Å]	1.03	0.98	1.02	0.98	0.97
	∡ _M−O−H_ [°]	106.5	108.9	95.0	123.2	118.4

d_M−O_=Bond distance between metal and oxygen. d_O−H_=Bond distance between oxygen and hydrogen. ∡ _M−O−<H_=angle between metal, oxygen, and hydrogen.

### 2.5 Electronic Properties of Hydroxylated Surfaces

**Figure 6:**
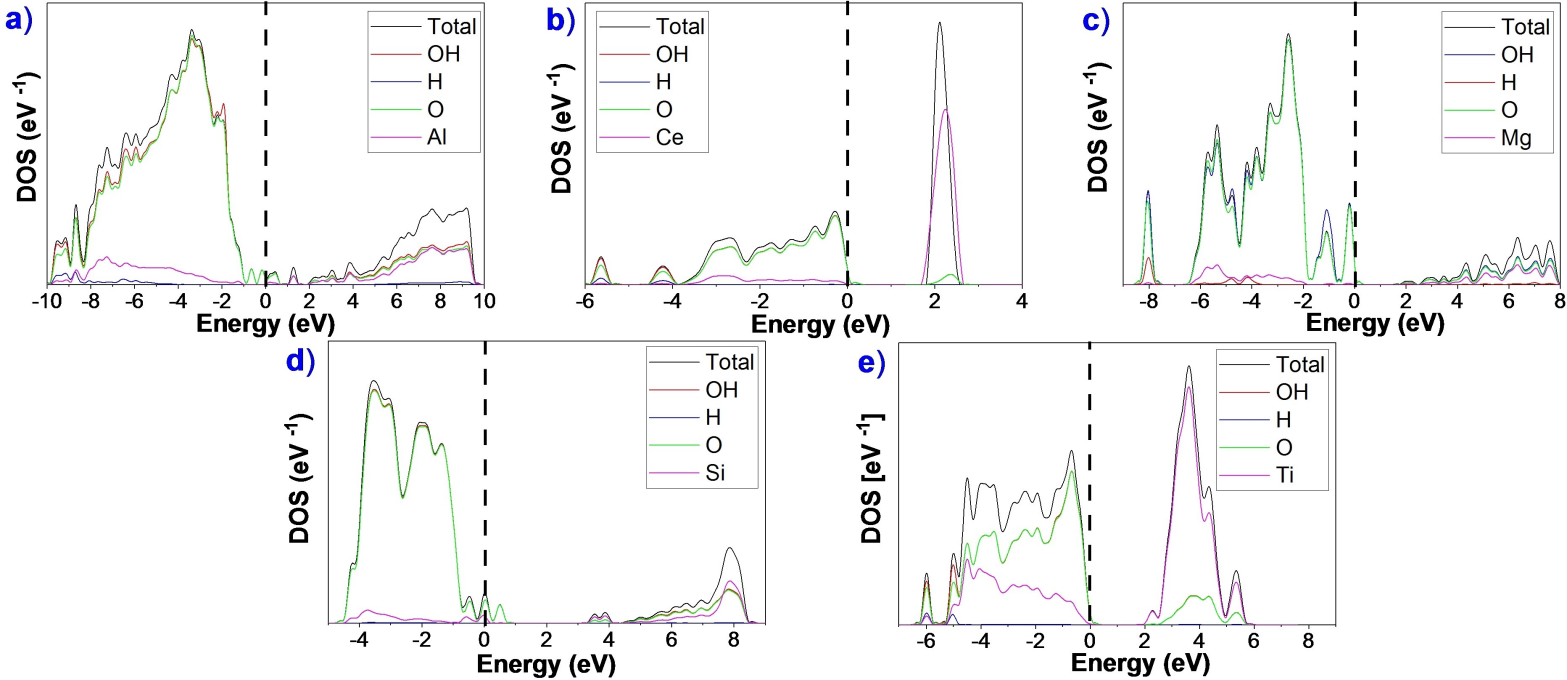
DOS and projected DOS on the metal, OH (I) and OH (II) for the hydroxylated surfaces: a) γ‐Al_2_O_3_ (110), b) CeO_2_ (111), c) MgO (100), d) β‐SiO_2_ (100), and e) a‐TiO_2_ (101).

Figure 6 shows the hydroxylated surfaces’ DOS and PDOS at the coverages summarised in Table 3. The appearance of new states in the V_B_ region compared with clean surfaces results from the OH groups partially saturating the surface's dangling bonds. Both C_B_ and V_B_ slightly change their relative position due to the O_2c_ and H bonding, which provokes an enlargement of the bands, becoming wider than in clean surfaces.^[62]^

The electronic structure indicates a change in the ionicity degree of the hydroxylated γ‐Al_2_O_3_ (110), CeO_2_ (111), β‐SiO_2_ (100) and a‐TiO_2_ (101) surfaces. The metal contributions to the V_B_ region suffer an upshift in energy, provoking an increase in the ionicity behaviour and a decrease of Lewis acidity. The protonation of the surfaces’ O also shifted the position of the O‐2p states.

The computational model allowed us to simulate the MgO (100) hydroxylation, which provokes the valence band edge to increase and reduce its bandgap. The two prominent peaks localised close to the Fermi level cause the Mg−O states to move towards lower energies and to decrease the degree of ionicity compared with the clean surface. The interaction of OH ions with Mg cations evokes MgOH groups’ generation, leading to a significant downshift of energy in Mg orbitals and an increase in acidity.^[63]^ As expected, β‐SiO_2_ (100) hydroxylation stabilises the structure and reduces the number of dangling bonds at the surface.^[64]^

### 2.6 Lewis Acid‐Base Descriptor

A common feature between oxide surfaces is their acid‐base properties, i. e. the metal cation acts as a Lewis acid site (electron acceptor), and the oxygen as a Lewis base site (electron donor). The overall acidity depends on the polarisation power from the cation and the anion.^[65]^ From the PDOS analysis of the unoccupied and occupied bands, one can derive the Lewis acidity (from the metal's states) and Lewis basicity (O‐2p states) to understand the oxygen and cation role in the oxide's reactivity. The V_B_ and C_B_ band centre (ϵ), defined in Equations (3) and (4), are proposed as Lewis acidity and basicity descriptors. Hence, the *ϵ_VB_* and *ϵ_CB_* are the band centre of the valence and conduction band respectively, *E_F_* is the Fermi energy, and *ρ(ϵ)* is the projected electronic density of state distribution on the orbitals of interest, i. e. *p, d, or f*.[^57a^, ^66^]

(3) $$\epsilon_{VB\;=}\frac{\int_{-\infty }^{E_{F}}\epsilon \cdot \rho \left(\epsilon \right)\;d\epsilon }{\underset{-\infty }{\int^{E_{F}}}\rho \left(\epsilon \right)\;d\epsilon }\;$$

(4) $$\epsilon_{CB\;=}\frac{\int_{E_{F}}^{\infty }\epsilon \cdot \rho \left(\epsilon \right)\;d\epsilon }{\underset{E_{F}}{\int^{\infty }}\rho \left(\epsilon \right)\;d\epsilon }$$

**Figure 7:**
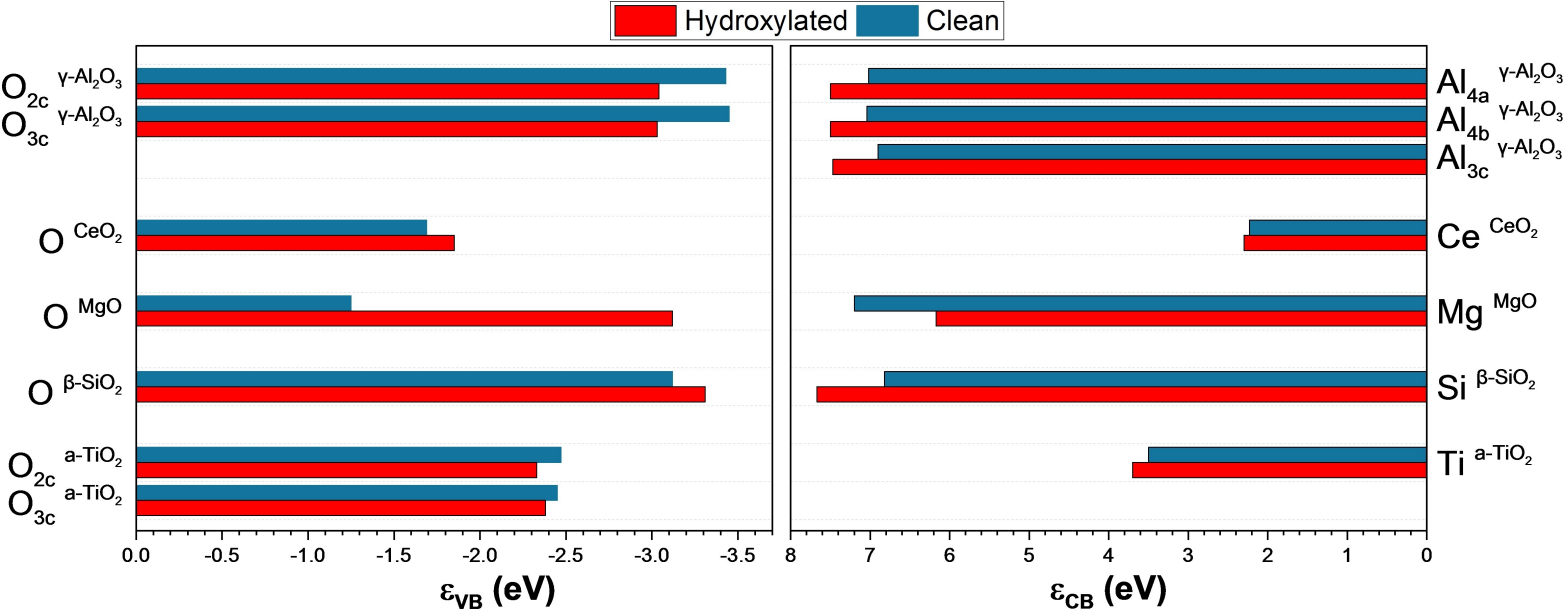
Band centres (ϵ) of the occupied (V_B_, left) and unoccupied states (C_B_, right) of the oxide surfaces in this study.

We summarised in Figure 7 the ϵ_VB_ and ϵ_CB_ collected from the projected band centres (Figure S5–S14). The ϵ_CB_ of the clean surfaces follow the order MgO>γ‐Al_2_O_3_>β‐SiO_2_>a‐TiO_2_>CeO_2_. However, the presence of d and f‐orbitals of Ti and Ce cation affects their ionic covalent character, as reported by Bordes‐Richard *et al*.,^[67]^ and trends including these oxides cannot be made. Comparing the Lewis acidity of the *sp* oxides, e. g. MgO, γ‐Al_2_O_3_ and β‐SiO_2_, we can categorise β‐SiO_2_ as an acid (ϵ_CB_=6.82 eV), γ‐Al_2_O_3_ as an amphoteric (ϵ_CB_=6.90 eV), and MgO as a base oxide (ϵ_CB_=7.20 eV). These results show that the lower the CB band centre, the higher the Lewis basicity. This reactivity agrees with the results from H and O adsorptions. The surfaces’ basicity is directly related to the 2p orbitals of the oxygen anion (ϵ_VB_). The ϵ_VB_ of the clean surfaces follow the order MgO>CeO_2_>a‐TiO_2_>β‐SiO_2_>γ‐Al_2_O_3_. According to these results, MgO has the highest Lewis basicity (ϵ_VB_=−1.25 eV), and γ‐Al_2_O_3_ has the lowest (ϵ_VB_=−3.45 eV), showing a distinct relation between these results and the Lewis acidity from the ϵ_CB_ results.

The surface hydroxylation impacts the Lewis acidity and basicity of the materials. Al_3c_ site in γ‐Al_2_O_3_ increases the energy of the conduction band with an Δϵ_CB_=0.57 eV (Δϵ, difference between the hydroxylated and clean surface), provoking a decrease in the Lewis acidity. Contrarily, the Lewis basicity of O atoms increases due to the oxygen V_B_ upshift.^[57a]^ On hydroxylated MgO (100), the interaction between the OH and Mg atoms causes an increase in the Lewis acidity strength (Δϵ_CB_=−1.03 eV) due to the decrease of the band centre (ϵ_CB_=6.17 eV). The band centre of the protonated O increases (ϵ_VB_=−3.12 eV), reducing the basicity of the surface (Δϵ_VB_=−1.87 eV). Similar trends are for CeO_2_ and a‐TiO_2_ with an Δϵ_CB_=0.07 and 0.20 eV, respectively. However, the surface protonation is different between these surfaces. The hydroxylation of CeO_2_ produces a downshift in the O orbitals energy (Δϵ_VB_=−0.16 eV), resulting in a decrease in the Lewis basicity linked with the cation's polarisation strength. On a‐TiO_2_, the surface hydroxylation increases the Lewis basicity on the O_2c_ site compared to the O_3c_ site. The hydroxylation of β‐SiO_2_ (100) surface also decreases its acidity (Δϵ_CB_=0.85 eV) and basicity (Δϵ_VB_=−0.19 eV) character due to the interaction of a Lewis acid‐base pair (OH^−^ and H^+^). However, the surface geometry impacts the reactivity because of their anion termination and lesser polarisation power than clean surfaces.^[24]^

### 2.7 Molecular Adsorption on Clean Oxide Surfaces

We brought the four lignol models represented in Figure 1, in addition to benzene (BEN) as a possible product of the HDO, close to four clean structures γ‐Al_2_O_3_ (110), CeO_2_ (111), MgO (100), and a‐TiO_2_ (101). Clean β‐SiO_2_ (100) was not included as it is always hydroxylated. We optimised three different molecular adsorption geometries according to the angle between the ring plane and the surface (90°, 45°, 0°), Table S6–S9. We placed the molecules according to our previous oxygen adsorption results, i. e., favouring the stronger affinity between the cation of the surface and the molecular oxygen group.

**Figure 8:**
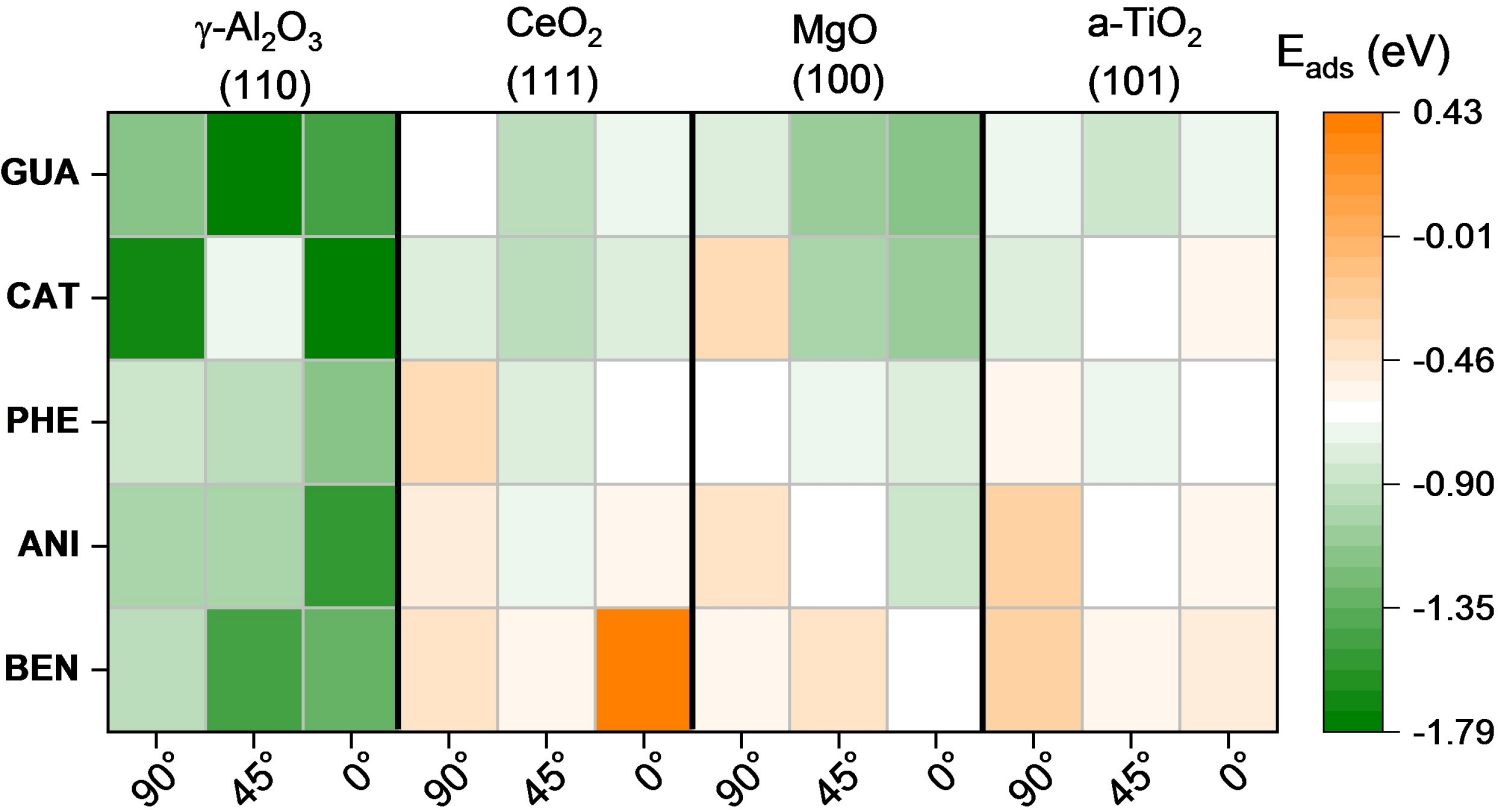
Calculated adsorption energy (E_ads_) of guaiacol (GUA), catechol (CAT), phenol (PHE), anisole (ANI) and benzene (BEN) with different orientations on clean oxide surfaces.

A heatmap in Figure 8 summarises the interaction energies resulting from the adsorption of the five compounds. Bader analysis and molecule distances to the surface are shown in Table S14–S17. The most favourable geometry modes for each molecule are included in Table 4. On γ‐Al_2_O_3_ (110), the flat orientation is generally preferred (at 0°) except for GUA and BEN, in which 45° orientation is 0.31 and 0.12 eV more favourable than the flat one. The surface of MgO preferably adsorbs the compounds parallel to the surface as it facilitates its interaction with the π‐conjugated orbitals of the molecule, which is more relevant than the interaction with the oxo groups. The O‐termination on CeO_2_ (111) and a‐TiO_2_ (101) structures prevent the interaction with the molecular oxo‐groups. On CeO_2_ (111), the 45° arrangement is the most favourable for all the compounds. It is the same on a‐TiO_2_ (101) except for CAT, in which OH groups favour the perpendicular orientation. Although we placed both groups (hydroxy and methoxy groups) closer to the cation atom at the surface, the methoxy group interacts weakly with the metal site, suggesting that the hydroxyl group is more accessible than the methoxy.^[68]^ The five compounds followed the adsorption strength trend (more negative) γ‐Al_2_O_3_ (110)>MgO (100)>CeO_2_ (111)>a‐TiO_2_ (101), as shown in Table 4.

**Table 4:** Calculated adsorption energies (E_ads_), distance molecular oxygen‐surface (d) and Bader charge (q) for the most favourable geometries modes on the oxide surfaces.

		γ‐Al_2_O_3_ (110)	CeO_2_ (111)	MgO (111)	a‐TiO_2_ (101)
**GUA**	initial angle [°]	45	45	0	45
	E_ads_ [eV]	−1.78	−0.91	−1.14	−0.85
	d‐O_1_ (−OH) [Å]	1.93	1.89	1.74	2.18
	d‐O_2_ (−OCH_3_) [Å]	2.10	1.91	1.98	2.31
	q [|e^−^|]	0.17	0.02	1.32	0.61
**CAT**	initial angle [°]	0	45	0	90
	E_ads_ [eV]	−1.75	−0.92	−1.12	−0.80
	d‐O_1A_ (−OH) [Å]	2.04	2.02	1.76	2.03
	d‐O_1B_ (−OH) [Å]	1.68	2.21	1.72	1.86
	q [|e^−^|]	0.47	0.31	0.45	0.16
**PHE**	initial angle [°]	0	45	0	45
	E_ads_ [eV]	−1.19	−0.79	−0.82	−0.71
	d‐O_1_ (−OH) [Å]	1.68	1.79	1.60	2.03
	q [|e^−^|]	0.49	0.02	0.16	0.53
**ANI**	initial angle [°]	0	45	0	45
	E_ads_ [eV]	−1.56	−0.69	−0.85	−0.63
	d‐O_2_ (−OCH_3_) [Å]	2.07	1.62	2.05	2.23
	q [|e^−^|]	0.34	0.03	0.24	0.42
**BEN**	Initial angle [°]	45	45	0	45
	E_ads_ [eV]	−1.45	−0.55	−0.64	−0.58
	q [|e^−^|]	0.06	0.02	0.12	0.02

### 2.8 Molecular Adsorption on Hydroxylated Surfaces

**Figure 9:**
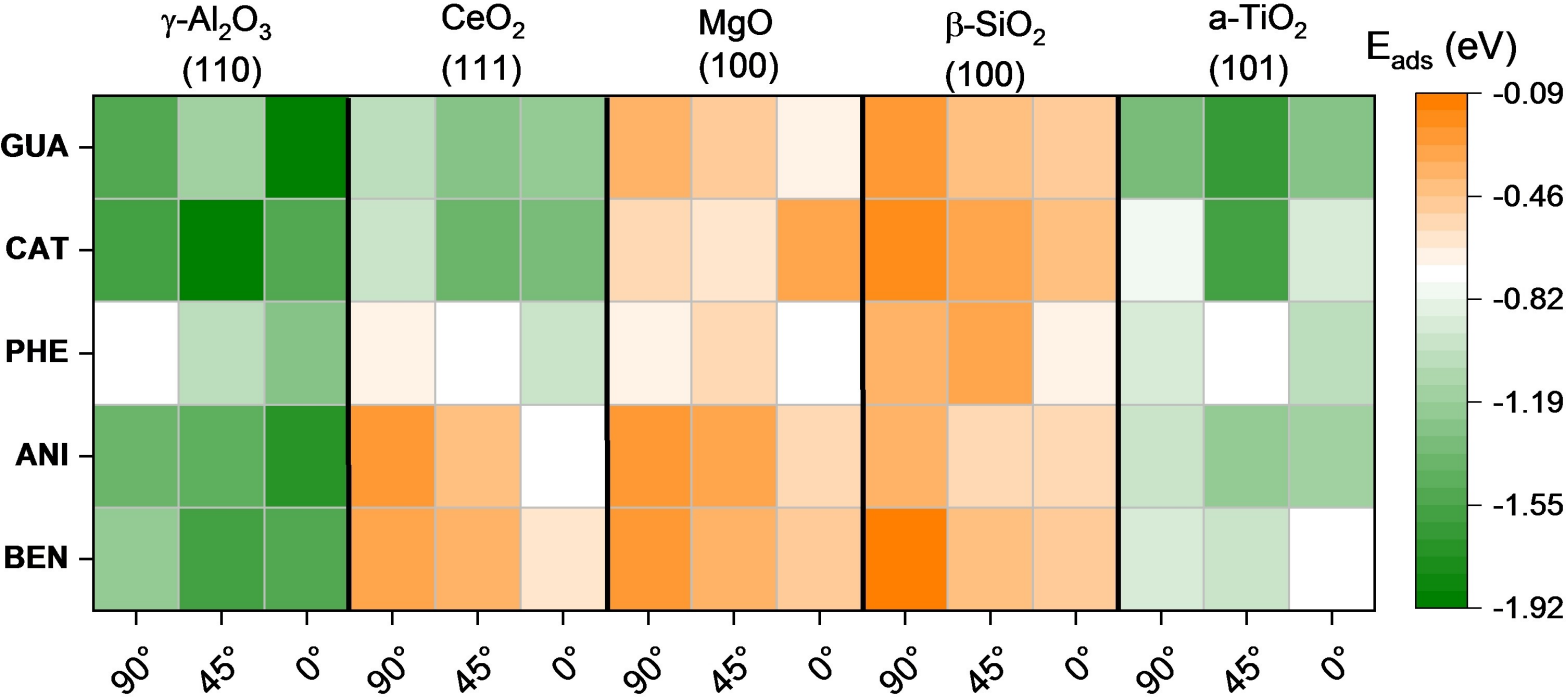
Calculated adsorption energy (E_ads_) of guaiacol (GUA), catechol (CAT), phenol (PHE), anisole (ANI) and benzene (BEN) with different geometry modes for hydroxylated oxide surfaces.

The adsorptions of lignin derivates on hydroxylated surfaces were carried out by placing the compounds nearby one of the surface hydroxyl groups, OH (I), and a neighbouring cation atom similarly to the initial geometry on the clean surfaces (see Tables S10–S13). Figure 9 summarises the interaction energies depending on the compound's initial orientations (90°, 45°, 0°). Bader analysis and distance between the molecules’ oxo‐groups and the surface are shown in Tables S18–S22. Table 5 summarises the properties of the most favourable adsorption modes on the hydroxylated surfaces. The majority of the compounds prefer absorbing parallel to the oxide surfaces maximising the interaction with the hydroxyl groups on the surface. Structures such as catechol have a strong affinity for hydrophilic surfaces because of their capacity to establish hydrogen bonds. Different experimental studies have confirmed the involvement of hydrogen bonding between the oxy‐compounds and the hydroxyl groups from the surface.^[69]^

**Table 5:** Calculated adsorption energies (E_ads_), distance molecular oxygen‐surface (d) and Bader charge (q) for the most favourable geometries modes on the hydroxylated surfaces.

		γ‐Al_2_O_3_ (110)	CeO_2_ (111)	MgO (100)	β‐SiO_2_ (100)	a‐TiO_2_ (101)
**GUA**	initial angle [°]	0	45	0	0	45
	E_ads_ [eV]	−1.91	−1.26	−0.69	−0.51	−1.62
	d‐O_1_ (−OH) [Å]	2.51	1.60	1.57	2.21	2.12
	d‐O_2_ (−OCH_3_) [Å]	2.30	2.30	2.38	2.22	1.95
	q [|e^−^|]	0.92	0.60	0.60	0.54	0.64
**CAT**	initial angle [°]	45	45	45	0	45
	E_ads_ [eV]	−1.89	−1.38	−0.61	−0.42	−1.59
	d‐O_1A_ (−OH) [Å]	1.78	1.67	1.63	2.26	1.63
	d‐O_1B_ (−OH) [Å]	1.89	1.65	1.51	2.11	1.97
	q [|e^−^|]	0.30	0.31	0.69	0.57	0.15
**PHE**	initial angle [°]	0	0	0	0	0
	E_ads_ [eV]	−1.26	−0.98	−0.75	−0.65	−1.05
	d‐O_1_ (−OH) [Å]	2.04	1.52	1.51	1.87	1.93
	q [|e^−^|]	0.32	0.48	0.27	0.40	0.63
**ANI**	initial angle [°]	0	0	0	0	45
	E_ads_ [eV]	−1.69	−0.74	−0.57	−0.54	−1.23
	d‐O_2_ (−OCH_3_) [Å])	2.02	2.28	2.24	1.94	2.18
	q [|e^−^|]	0.27	0.08	0.14	0.58	0.40
**BEN**	initial angle [°]	45	0	0	0	45
	E_ads_ [eV]	−1.61	−0.61	−0.50	−0.50	−0.96
	q [|e^−^|]	0.39	0.04	0.06	0.84	0.32

For γ‐Al_2_O_3_ (110), the model compounds interaction with hydroxylated surfaces is slightly more favourable than with clean surfaces although both expose the Al_3c_ site. The increase of O atoms’ basicity upon hydroxylation (Δϵ_VB_=0.42 eV) creates new labile Al−O pairs. Hence, GUA presents a stronger interaction with the hydroxylated γ‐Al_2_O_3_ at 0° (E_ads_=−1.91 eV) compared to the pristine surface at 45° (E_ads_=−1.78 eV). The most notable increase is with ANI, strengthening the interaction between the hydroxylated surface and the molecule by around 8.3 % (E_ads_=−1.69 eV). These results conclude that the OH and H's distribution over the clean surface does not block Al sites and strengthen the hydrogen bonds with the π‐system of the oxy‐compounds.[^57a^, ^70^] Similar results were found for CeO_2_ (111), where the compounds’ interactions with the hydroxylated CeO_2_ is slightly stronger than the pristine surface (by ∼19 %). These results suggest that the incorporation of OH and H on CeO_2_ improves the interaction at low hydroxyl coverage with a minimal decrease in acidity (Δϵ_CB_=0.07 eV) and a moderate reduction of basicity (Δϵ_VB_=−0.16 eV). All the compounds prefer the parallel orientation with the surface except for GUA and CAT that remain at ∼45°.

The most dramatic increases of interaction with the oxy‐compounds are seen for hydroxylated a‐TiO_2_ (101). Like CeO_2_, the hydroxylated a‐TiO_2_ did not show a considerable difference in the acid/base properties. Its Lewis acid strength decreases (Δϵ_CB_=0.20 eV) and its Lewis basicity increases (Δϵ_VB_=0.14 eV). These results suggest that Ti d‐orbitals metal oxide's interaction with HOMO from the molecule is stronger than on p‐oxides. For example, ANI has the highest adsorption orientation at 45° (E_ads_=−1.23 eV), indicating that the methoxy group interacts strongly with the surface.^[71]^ The lack of trends between charge transfer and adsorption energy suggests that the surface terminal hydroxyl groups significantly impact the interaction with the aromatic ring and the molecule's oxo group(s). Previous studies have indicated that the active sites of TiO_2_ are strictly linked to the contact of water, favouring the direct deoxygenation mechanism of phenolic compounds due to the cleavage of the C−OH bond.^[72]^

Upon hydroxylation, β‐SiO_2_ (100) leads to the formation of germinal silanol groups, Si‐(OH)_2_, with reduced the acid character (Δϵ_VB_=−0.19 eV) and providing new adsorption sites.^[51i]^ The most favourable interaction between the surface and lignols is at 0°, exposing the phenyl ring to the hydroxyl groups. For example, in CAT, the highest adsorption energy is at 0° with an E_ads_=−0.42 eV, while the weaker interaction is through the OH groups, E_ads_=−0.21 eV. The hydroxyl groups act as new active sites that create long‐range hydrogen bonds with the π‐system of the model compounds.^[73]^

**Figure 10:**
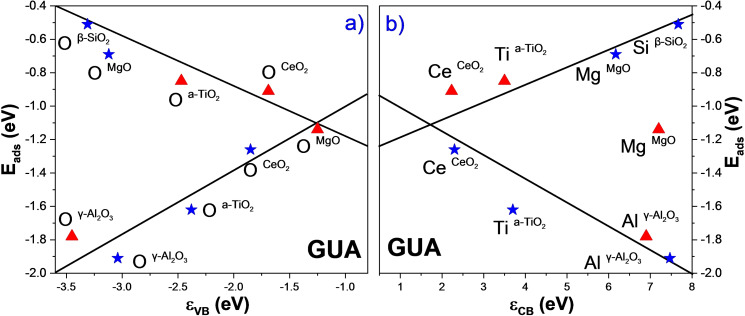
Adsorption energies for the model compounds versus band centres (ϵ_VB_ and ϵ_CB_) for the five oxide surfaces (clean and hydroxylated). Colour code: Red triangle (▴) and blue star (★) represent the clean and the hydroxylated oxide surfaces, respectively. Black trend lines are inset to guide the eye.

MgO (100) is the only oxide examined that, upon hydroxylation, reduces its affinity to interact with the phenolic compounds between 9 %–46 %. CAT is the most notorious case among all the compounds studied. These results can be explained because the clean MgO basicity (100) is lower than the hydroxylated surface (Δϵ_VB_=−1.87 eV).^[74]^ Figure 10 shows the relation of the adsorption energies with the valence (ϵ_VB_) and conduction (ϵ_CB_) band centres, i. e. the acid‐base properties. As expected, a clear trend can be seen between the base sites and the compounds, but not with acid sites (Mg cation), meaning that the Lewis basicity controls the interaction with the compounds. The results confirm that the hydroxyl groups shield the adsorption sites of the surface (base site), which weakens their adsorption capabilities.

Figure 10 and Figure S15 display the trends between the oxides’ acid‐base properties and the adsorption energies of the phenolic groups, which combined with the oxophilicity in section c, can guide the HDO catalysts support selection. The ϵ_VB_ linear trend, related to the occupied O‐2p orbitals of the surfaces makes, is a good descriptor based on the Lewis basicity properties. The trends confirm that a decrease in basicity character strengthens the interaction with the phenolic compounds. Similarly, ϵ_CB_ is linearly related to the adsorption energy, although less reliable when including d‐ and f‐orbitals, further modifying the oxides’ ionic‐covalent character.^[67]^

## 3 Conclusions

During the catalytic hydrodeoxygenation (HDO) process of pyrolysed lignin, the catalyst support is no innocent, and it should be selected carefully. We have investigated the interaction of five different compounds derived from lignin, e. g., guaiacol (GUA), catechol (CAT), phenol (PHE), anisole (ANI) and benzene (BEN), on clean and hydroxylated oxide surfaces using accurate periodic DFT study methods (GGA‐RPBE). The oxide surfaces studied include acid‐base and reducible properties, e. g., γ‐Al_2_O_3_ (110), CeO_2_ (111), MgO (100), β‐SiO_2_ (100) and a‐TiO_2_ (101) and have been suggested as candidates to support HDO catalysts. We examined the electronic structure of the clean and hydroxylated surfaces. For CeO_2_ (111), β‐SiO_2_ (100), γ‐Al_2_O_3_ (110) and a‐TiO_2_ (101), the effect of the surface hydroxylation process slightly increases the adsorption strength with all the compounds studied. The molecules generally adopt a parallel orientation with the surface, maximising the interaction between the molecular π‐system and the dangling orbitals at the surface. These results confirm that the interaction with the oxy compounds behaves differently for clean and hydroxylated oxide surfaces. We introduced the conduction and valence band centres as Lewis acid/base properties descriptor, providing a precise clarification of the acid, amphoteric and base behaviours. Besides it, the electronic structure provided insights into surfaces’ ionic/covalent character. Although the p‐oxides, e. g., γ‐Al_2_O_3_, β‐SiO_2_ and MgO, are not directly comparable with CeO_2_ and a‐TiO_2_ due to d‐ and f‐bands participation, the results confirmed that γ‐Al_2_O_3_ (110) is the support with a higher affinity to oxygen and all the oxygen compounds due to its high Lewis acidity. Hence, there are strong indications that the bond strength is a good descriptor for the selection of oxide supports. An oxide‐support interaction that is neither too weak nor too strong will avoid high activation barriers and low reactivity. Although weak Lewis acidity supports are favourable to avoid coke formation, there are limitations between the surface bonding and the studied descriptors to predict the reaction rate for undesired reactions.

## Conflict of interest

The authors declare no conflict of interest.

## Supporting information

As a service to our authors and readers, this journal provides supporting information supplied by the authors. Such materials are peer reviewed and may be re‐organized for online delivery, but are not copy‐edited or typeset. Technical support issues arising from supporting information (other than missing files) should be addressed to the authors.

Supporting Information
