# Supplementary material for: The Effect of Pristine and Hydroxylated Oxide Surfaces on the Guaiacol HDO Process: A DFT Study
Source: Chemphyschem. 2021 Sep 30;23(1):e202100583. doi: 10.1002/cphc.202100583 (PMC9292963; doi:10.1002/cphc.202100583)
Supplement: Supplementary file 1 — Supporting Information [file CPHC-23-0-s001.pdf]

ChemPhysChem

Supporting Information

The Effect of Pristine and Hydroxylated Oxide Surfaces on the Guaiacol HDO Process: A DFT Study

Fabian Morteo-Flores and Alberto Roldan*

CONTENT:

1. Bulk calculations
2. Test convergence
3. Total and projected density of states (DOS and PDOS)
4. Hydrogen and oxygen adsorption
5. Band centre (ε)
6. Adsorption on clean and hydroxylated surfaces (Illustrations)
7. Model compounds adsorption on clean surfaces
8. Model compounds adsorption on hydroxylated surfaces
9. Adsorption energies for the model compounds versus band centres
10. References

1. Bulk calculations

We performed preliminary test calculations were performed for five different oxide supports: γ-Al_2O_3, CeO_2, MgO, β-SiO_2 and anatase TiO_2 (a-TiO_2); bulk structures can be seen in ***Figure S1***. We have carried out spin-polarised density functional theory (DFT). Moreover, the exchange and correlation contributions were calculated using the generalised gradient approximation (GGA) with the revised functional of Perdew-Burke-Ernzerhof (RPBE) to obtain the best computational setting, such as k-points and cut-off energy.[1] Studies showed that an E_{cut} of 550 eV and a number of k-points greater than 11 k-points were accurate enough to reach the plateau **(*Figure S2-a*** and ***S2-b*).**[2]

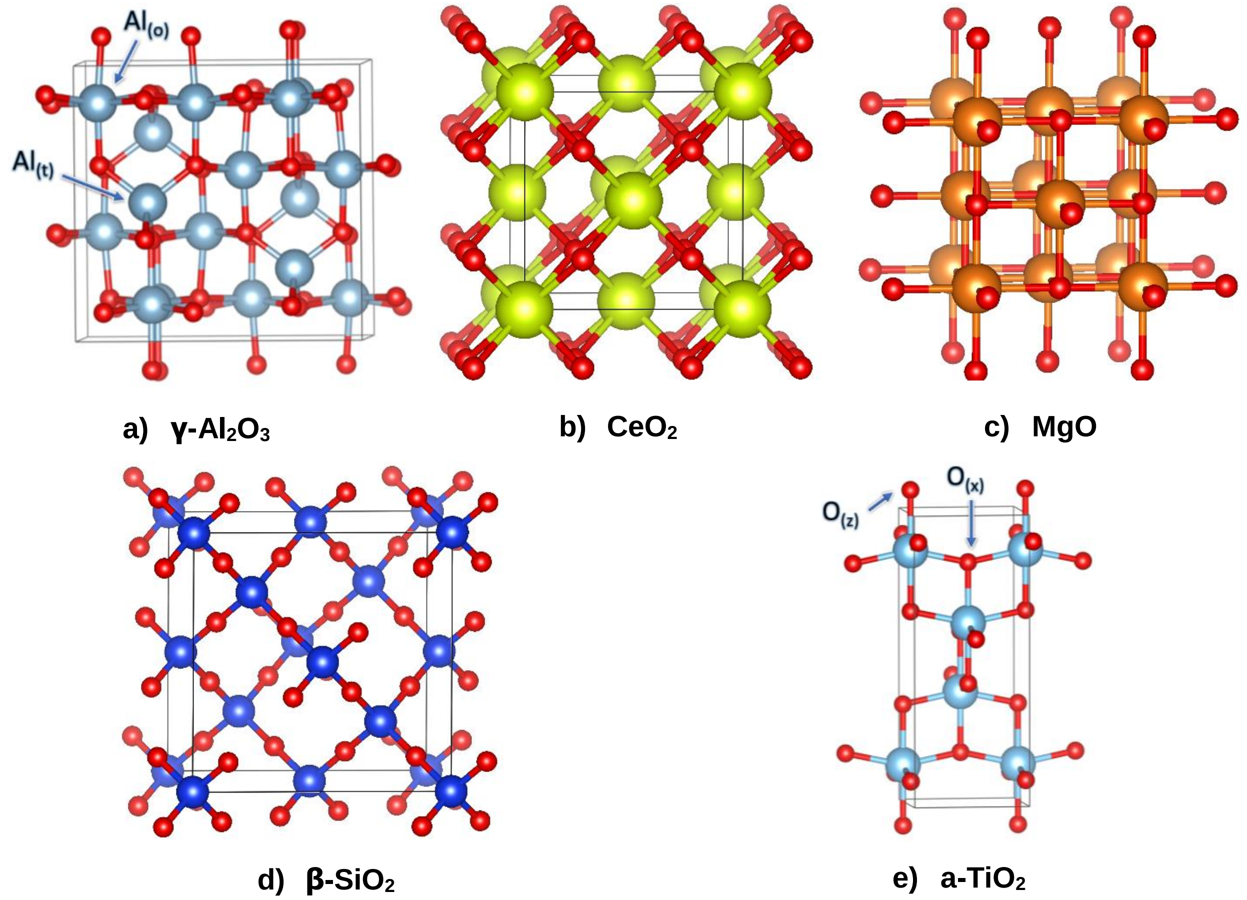

Figure S1. *Bulk structure of a) Digne model-γ-Al_2O_3, b) CeO_2, c) MgO, d) β-cristobalite SiO_2 and e) TiO_2 anatase used for this study.*

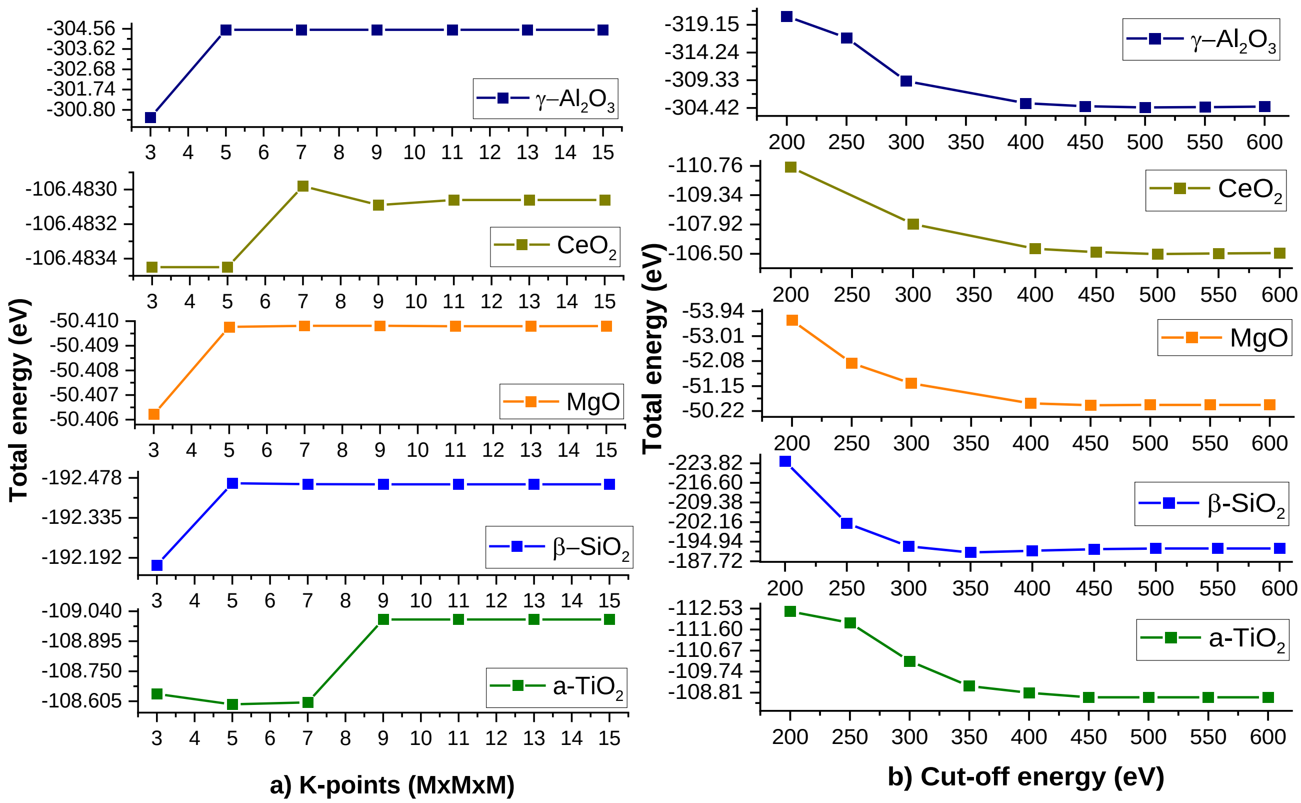

Figure S2. *The convergence of total energy (a) K-points and b) cut-off energy of the bulk structures*

Based on the previous tests, we performed calculations to get the optimised lattice parameters for the materials. For SiO_2 and γ-Al_2O_3, we used the ideal β-cristobalite structure with cubic Fd3m symmetry and the Digne non-spinel, respectively.[3] In ***Table S1***, the results for the optimised lattice parameter (a_o, Å) are presented for the oxide bulk structures. Their structure properties, such geometry and distances between atoms (Metal-oxygen, oxygen-oxygen) were compared with other works with a small error margin.

Table S1. *Calculated properties for the bulk structures of oxide surfaces a) lattice parameters and b) M-O and O-O distances (in Å)*

		a_o (Å)	d_{M-O} (Å)	d_{o-o} (Å)
γ-Al_2O_3	**This work**	a = 5.57,<br>c = 8.069	Al(o) = 1.91,<br>Al(t) = 1.76	2.62
	**Other works**	a = 5.66 [4],<br>c= 8.08 [5]	Al(o) = 1.94 [6],<br>Al(t) = 1.78 [6]	2.70
	**Error (%)**	a =1.63%,<br>c = 0.14%	Al(o) = 1.55%,<br>Al(t) = 1.12%	2.96%
CeO_2	**This work**	5.47	2.37	2.73
	**Other works**	5.44	2.36 [7]	2.70 [8]
	**Error (%)**	0.63%	0.34%	1.11%
MgO	**This work**	4.22	2.10	2.98
	**Other works**	4.21 [9]	2.13 [10]	2.70 [8]
	**Error (%)**	0.17%	1.22%	9.39%
β-SiO_2	**This work**	7.47	1.62	2.641
	**Other works**	7.45 [8]	1.61 [8]	2.70 [8]
	**Error (%)**	0.23%	0.25%	2.19%
a-TiO_2	**This work**	a = b = 3.83<br>c = 9.62	O_x =1.96<br>O_z = 2.00	d_{M-M} = 3.83
	**Other works [11]**	a = b = 3.79<br>c = 9.74	O_x = 1.94<br>O_z = 2.00	d_{M-M} = 3.82
	**Error (%)**	a = b = 1.08%<br>c = 1.20 %	O_X = 0.75%<br>O_Z= 0.25%	d_{M-M} = 0.18 %

Al(o), Al(t) = Al octahedral and tetrahedral respectively. * dM-O, dO-O, dM-M = Distance of O and the metal (M), oxygen (O) or metal (M) respectively.

2. Test convergence

We performed energy convergence tests on the number of layers, vacuum thickness, and k-points (see ***Figure S3***) to find the best compromise between accuracy and computational cost.[12] METADISE was used to create the structure in different orientations: (100), (101), (110), (111). In this case, we chose the (100) direction to represent the study of the five different oxides supports. The Hubbard correction (DFT+U) was applied on the Ce surface to locate the 4f-orbitals with the following parameters: U_{eff} = 4eV (U=5 eV and J=1 eV) for CeO_2.[13]

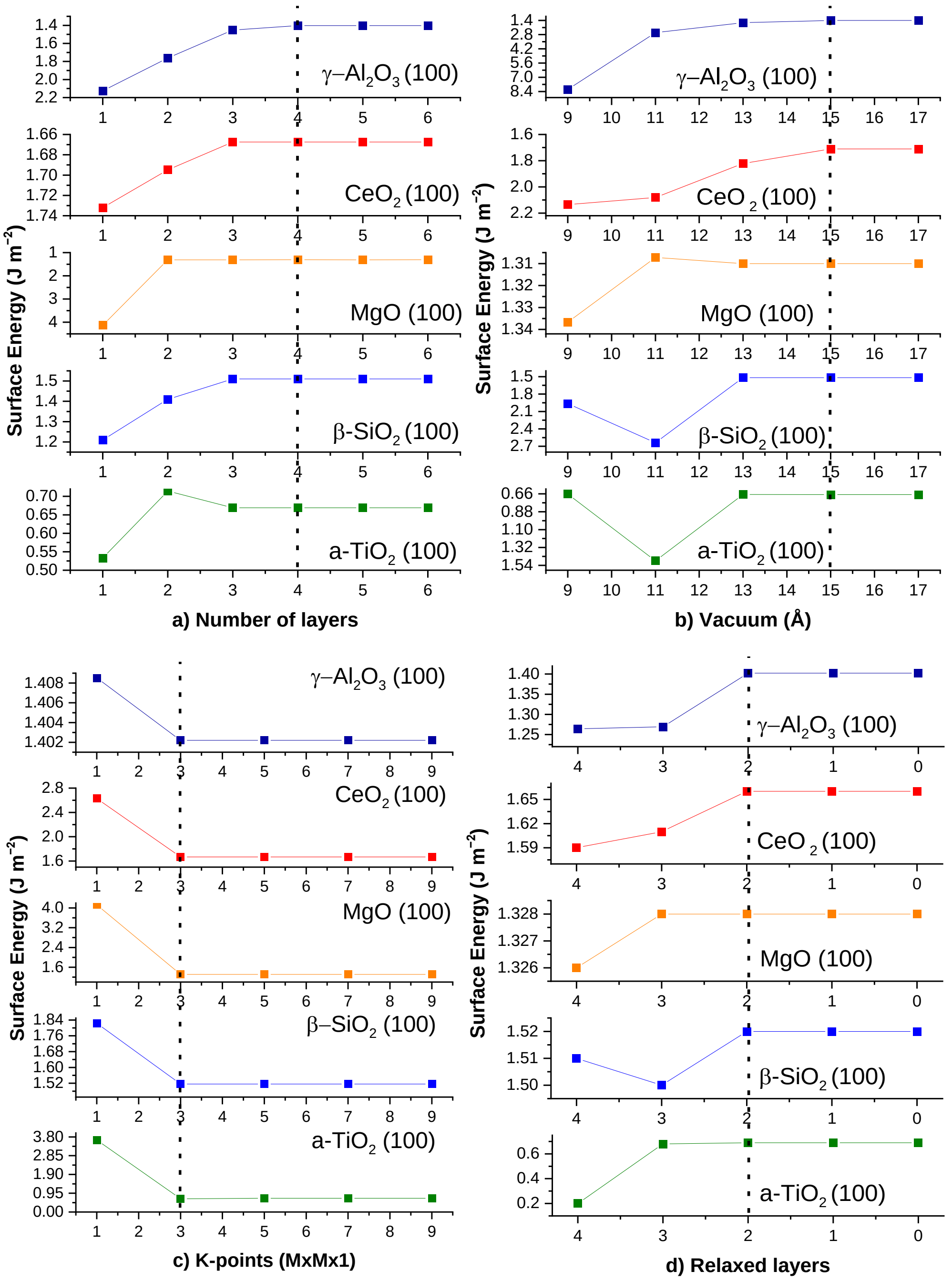

Figure S3: Variation of surface energy of (a) number of layers, (b) vacuum thickness, and (c) number of k-points for γ-Al_2O_3, CeO_2, MgO, β-SiO_2, and a-TiO_2 (100) facet

3. Total and projected density of states (DOS and PDOS)

The density of states (DOS) and projected density of states (PDOS) of metal and oxygen of the oxides' surfaces are shown in ***Figure S4***. Calculated bandgaps of surfaces and bulk of oxide surfaces are shown in ***Table S2***.

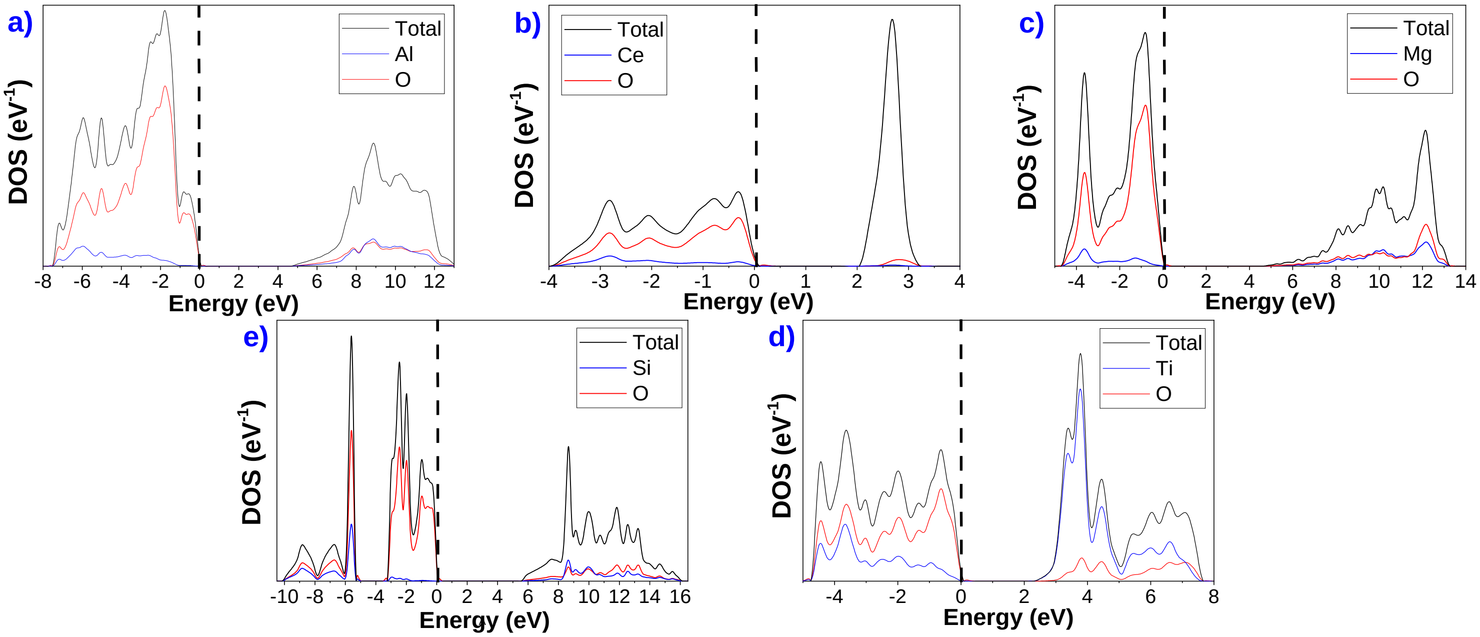

Figure S4*. DOS and projected DOS for bulk a) γ-Al_2O_3, b) CeO_2, c) MgO, d) β-SiO_2, and a-TiO_2. DOS are given in eV^{-1}.*

4. Hydrogen and oxygen adsorption

The following tables (***Table S3-S4***) summarized the hydrogen and oxygen adsorption energies for the five oxides surfaces in different adsorption sites (T-top, B-bridge, H-hollow). Bader charge study was carried out to determine the interaction between the atom (hydrogen and oxygen) with the surface.

Table S3: *Calculated hydrogen adsorption energies (E_{ads}), Bader charge analysis (q) and distance between hydrogen - surface (d_H) for the oxide surfaces.*

		T_1 (O)†	T_2 (M)†	B (M-O)	H
**γ-Al_2O_3 (110)**	**E_{ads} (eV)**	−1.31 – T_{1a} (O_{2c})<br>−0.60 – T_{1b} (O_{3c})	2.15 – T_{2a} (Al_{4a})<br>1.42 – T_{2b} (Al_{4b})<br>0.13 – T_{2c} (Al_{3c})	−0.66	2.20
	**q |e⁻|**	0.70 – T_{1a} (O_{2c})<br>0.67 – T_{1b} (O_{3c})	−0.64 – T_{2a} (Al_{4a})<br>−0.29 – T_{2b} (Al_{4b})<br>−0.06 – T_{2c} (Al_{3c})	0.61	−0.95
	**d_H (Å)**	0.97 – T_{1a} (O_{2c})<br>0.97 – T_{1b} (O_{3c})	1.60 – T_{2a} (Al_{4a})<br>1.61 – T_{2b} (Al_{4b})<br>1.58 – T_{2c} (Al_{3c})	1.78	1.00
**CeO_2 (111)**	**E_{ads} (eV)**	−1.15	2.20	-0.47	−0.79
	**q |e⁻|**	0.60	0.02	0.58	0.61
	**d_H (Å)**	0.97	2.95	0.99	0.99
**MgO (100)**	**E_{ads} (eV)**	−0.16	2.16	−0.08	−0.07
	**q |e⁻|**	0.63	0.64	−0.20	0.64
	**d_H (Å)**	1.33	2.32	2.11	1.51
**β-SiO_2 (100)**	**E_{ads} (eV)**	−1.00	- - - **	−0.81	0.63
	**q |e⁻|**	0.66	- - - **	0.69	−0.59
	**d_H (Å)**	0.97	- - - **	1.06	1.49
**a-TiO_2 (101)**	**E_{ads} (eV)**	−0.10 – T_{1a} (O_{2c})<br>0.69 – T_{1b} (O_{3c})	2.18	2.22 – B_1 (Ti-O_{2c})<br>2.19 – B_2 (Ti-O_{3c})	2.26
	**q |e⁻|**	0.07 – T_{1a} (O_{2c})<br>0.05 – T_{1b} (O_{3c})	−0.34	0.02 – B_1 (Ti-O_{2c})<br>−0.03 – B_2 (Ti-O_{3c})	0.02
	**d_H (Å)**	0.97 – T_{1a} (O_{2c})<br>0.98 – T_{1b} (O_{3c})	2.52	2.49 – B_1 (Ti-O_{2c})<br>2.41 – B_2 (Ti-O_{3c})	2.48

† T_1 (O) = Interaction between the hydrogen atom and the oxygen atom from the surface. T_2 (M) = Interaction between the hydrogen atom and the metal atom from the surface.

** Not found.

Table S4: *Calculated oxygen adsorption energies (E_{ads}), Bader charge analysis (q) and distance between hydrogen - surface (d_o) for the oxide surfaces.*

		T_1 (O)†	T_2 (M)†	B (M-O)	H
**γ-Al_2O_3 (110)**	**E_{ads} (eV)**	- - - T_{1a} (O_{2c}) **<br>- - - T_{1b} (O_{3c}) **	−0.39 – T_{2a} (Al_{4a})<br>−2.02 – T_{2b} (Al_{4b})<br>−2.30 – T_{2c} (Al_{3c})	−1.11	−0.09
	**q |e⁻|**	- - - T_{1a} (O_{2c}) **<br>- - - T_{1b} (O_{3c}) **	−0.59 – T_{2a} (Al_{4a})<br>−1.18 – T_{2b} (Al_{4b})<br>−1.41 – T_{2c} (Al_{3c})	−0.48	−0.43
	**d_O (Å)**	- - - T_{1a} (O_{2c}) **<br>- - - T_{1b} (O_{3c}) **	1.87 – T_{2a} (Al_{4a})<br>1.88 – T_{2b} (Al_{4b})<br>1.76 – T_{2c} (Al_{3c})	1.60	1.88
**CeO_2 (111)**	**E_{ads} (eV)**	- - - **	−1.42	−1.41	−1.44
	**q |e⁻|**	- - - **	−0.47	−0.46	−0.52
	**d_O (Å)**	- - - **	1.90	1.40	1.32
**MgO (100)**	**E_{ads} (eV)**	−0.96	0.40	−0.57	−0.23
	**q |e⁻|**	−0.74	−0.27	−0.69	−0.71
	**d_O (Å)**	1.53	2.01	1.61	2.05
**β-SiO_2 (100)**	**E_{ads} (eV)**	0.50	- - - **	−0.48	0.27
	**q |e⁻|**	0.01	- - - **	−0.75	−0.23
	**d_O (Å)**	1.35	- - - **	1.64	1.85
**a-TiO_2 (101)**	**E_{ads} (eV)**	0.14 – T_{1a} (O_{2c})<br>0.47 – T_{1b} (O_{3c})	0.28	−0.96 – B_1 (Ti-O_{2c})<br>−0.50 – B_2 (Ti-O_{3c})	0.56
	**q |e⁻|**	−0.32 – T_{1a} (O_{2c})<br>−0.39 – T_{1b} (O_{3c})	−0.39	−0.34 – B_1 (Ti-O_{2c})<br>−0.36 – B_2 (Ti-O_{3c})	−0.36
	**d_O (Å)**	1.33 – T_{1a} (O_{2c})<br>1.50 – T_{1b} (O_{3c})	1.50	1.44 – B_1 (Ti-O_{2c})<br>1.35 – B_2 (Ti-O_{3c})	1.95

† T_1 (O) = Interaction between the oxygen atom and the oxygen atom from the surface. T_2 (M) = Interaction between the oxygen atom and the metal atom from the surface.

** Not found.

5. Band centre (ε)

Figures S5-S14 show PDOS of the occupied (ε_{VB}) and unoccupied states (ε_{CB}) for clean and hydroxylated surfaces.

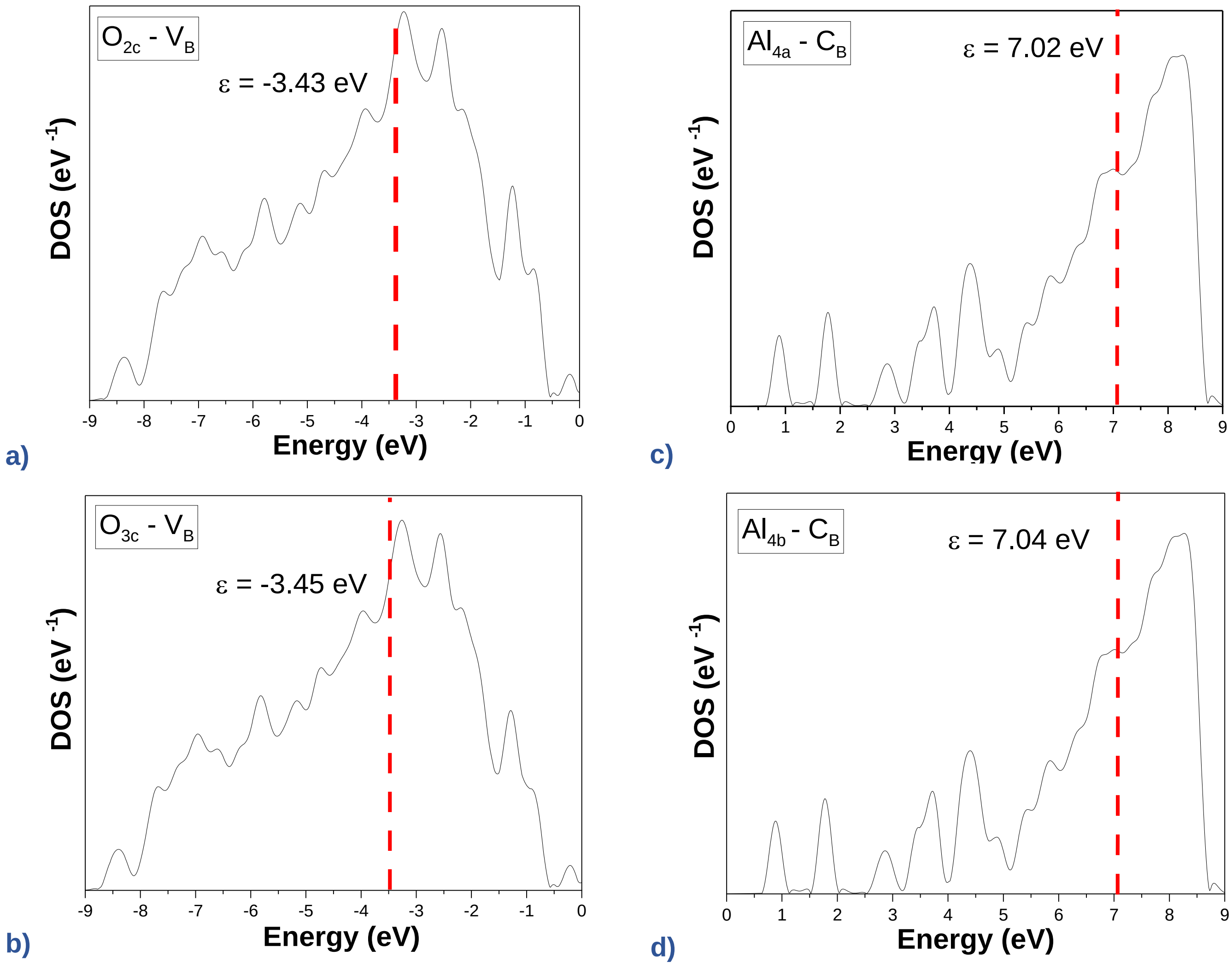

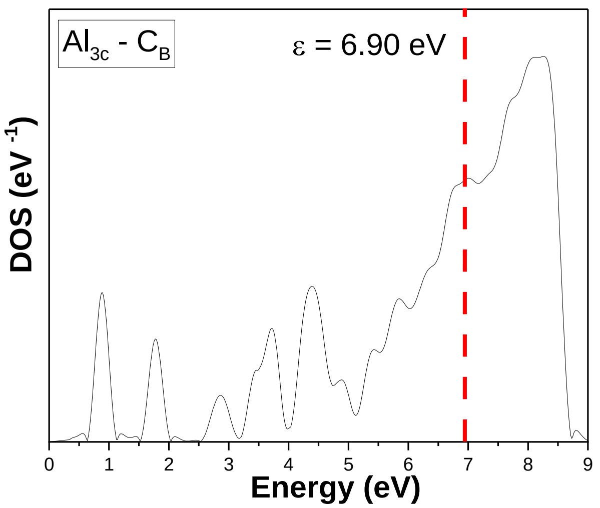

Figure S5. *PDOS for O-2p states for a) O_{2c} and b) O_{3c}; and PDOS for Al 3p states for c) Al_{4a}, d) Al_{4b} and Al_{3c} for clean γ-Al_2O_3 (110). Red dashed line represents the band centre in eV.*

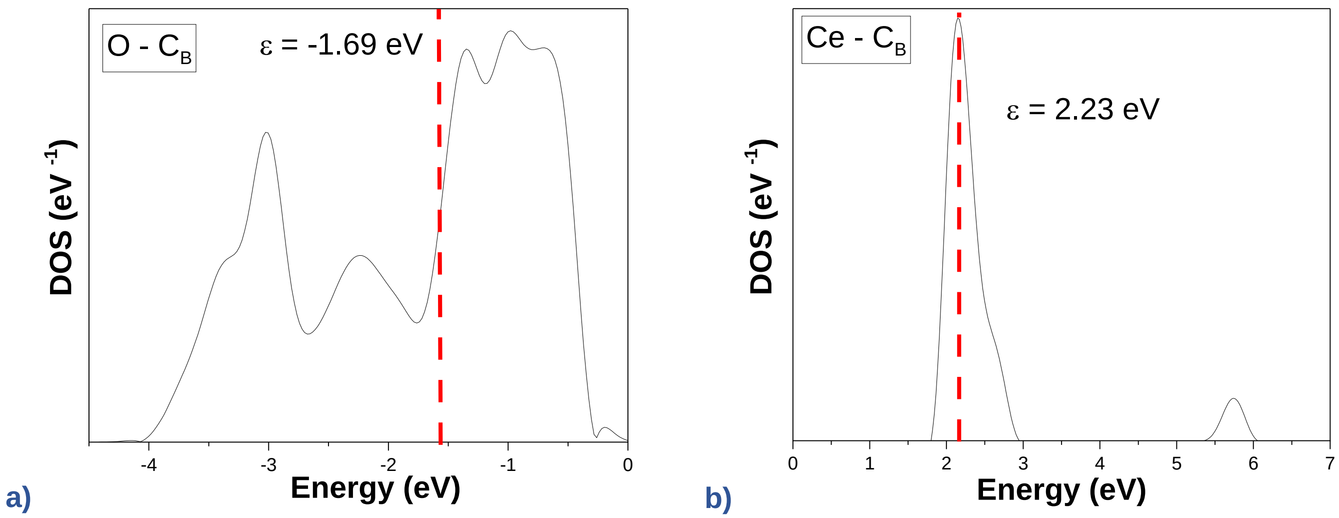

Figure S6. *PDOS for O-2p states for a) O; and PDOS for Ce 4f, 5d and 6s states for c) Ce for clean CeO_2 (111). Red dashed line represents the band centre in eV.*

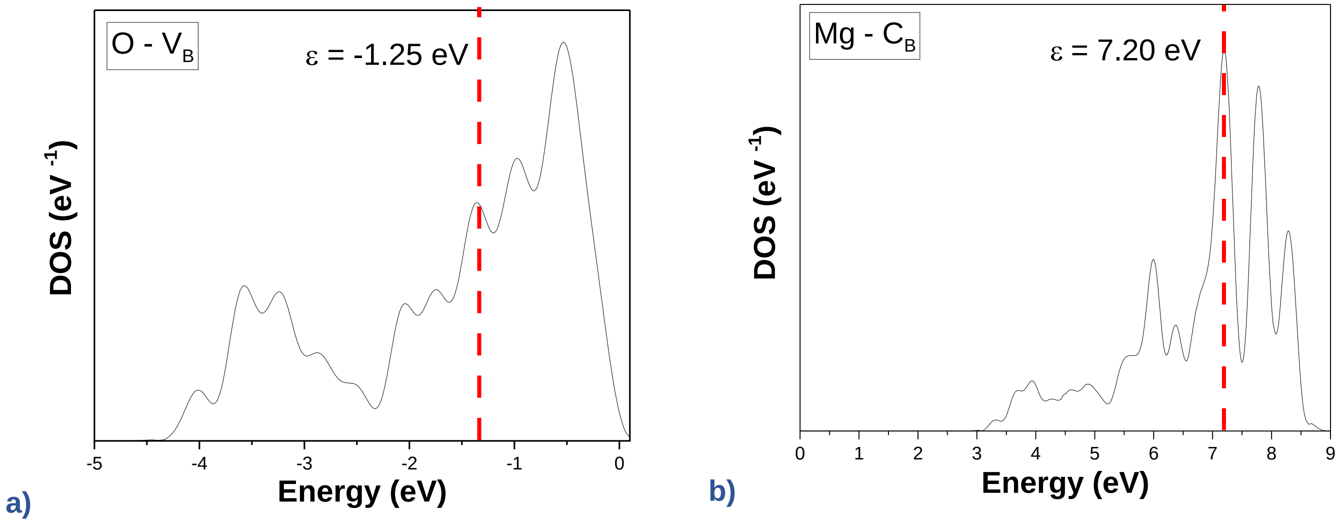

Figure S7. *PDOS for O-2p states for a) O; and PDOS for Mg-3s states for c) Mg for clean MgO (100). Red dashed line represents the band centre in eV.*

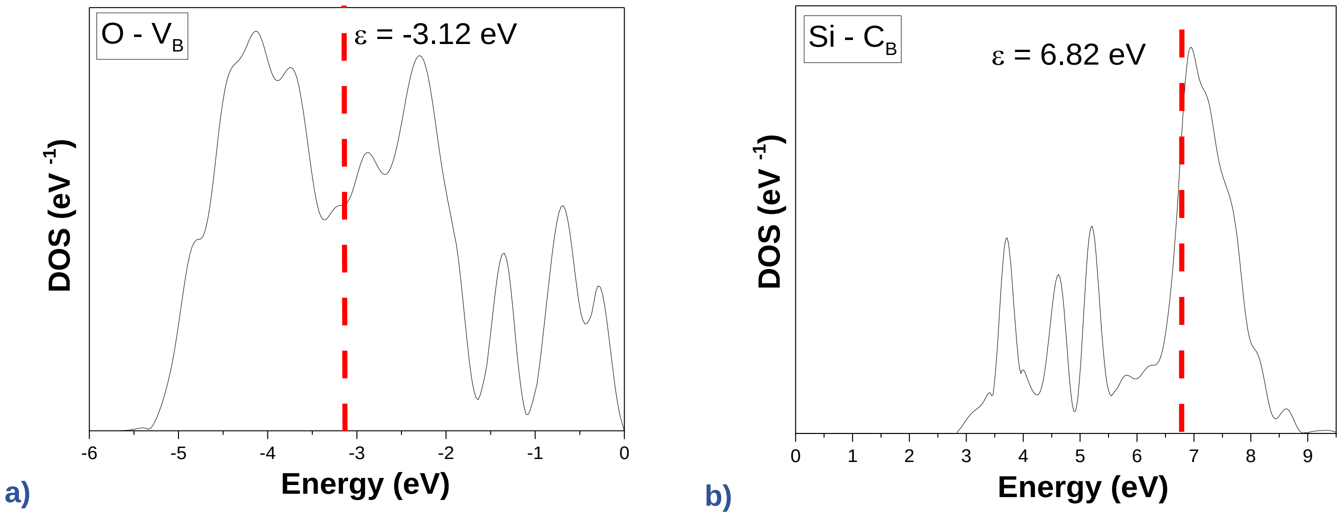

Figure S8. *PDOS for O-2p states for a) O; and PDOS for Si 3p states for c) Si for clean β-SiO_2 (100). Red dashed line represents the band centre in eV.*

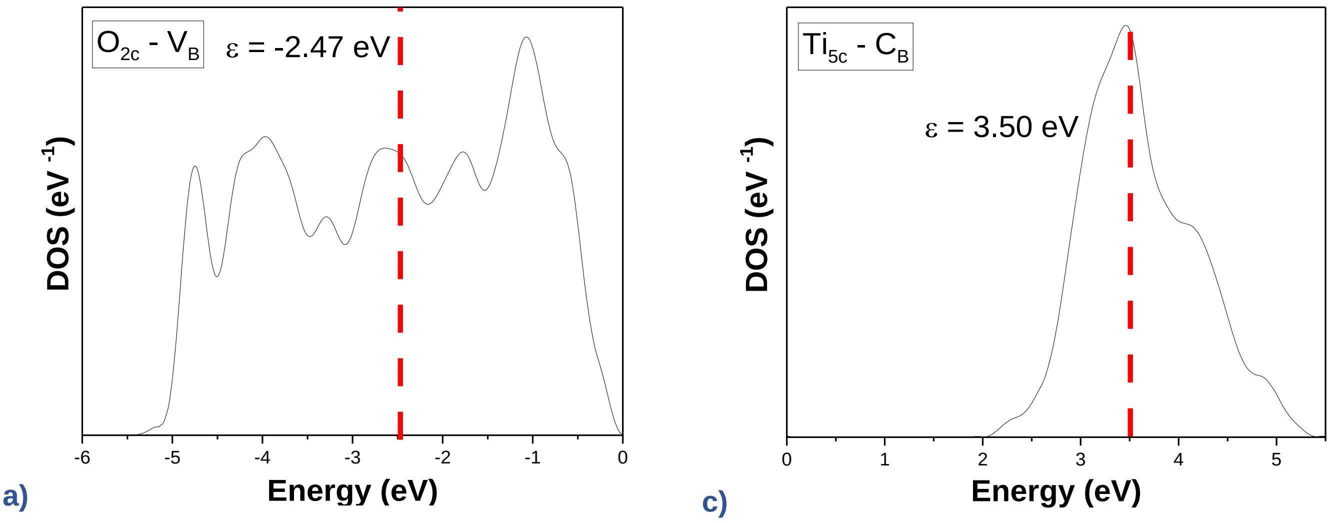

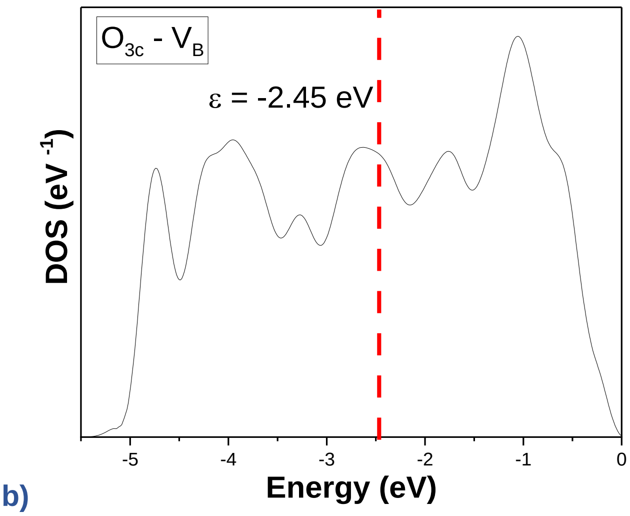

Figure S9. *PDOS for O-2p states for a) O_{2c} and b) O_{3c}; and PDOS for Ti 3d and 4p states for c) Ti for clean a-TiO_2 (101). Red dashed line represents the band centre in eV.*

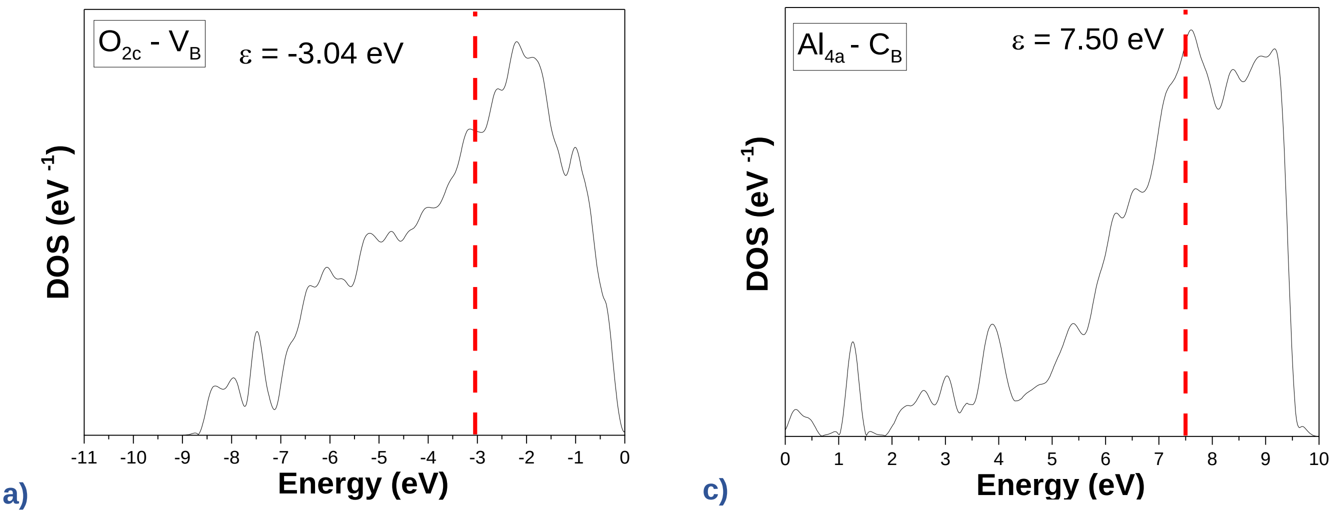

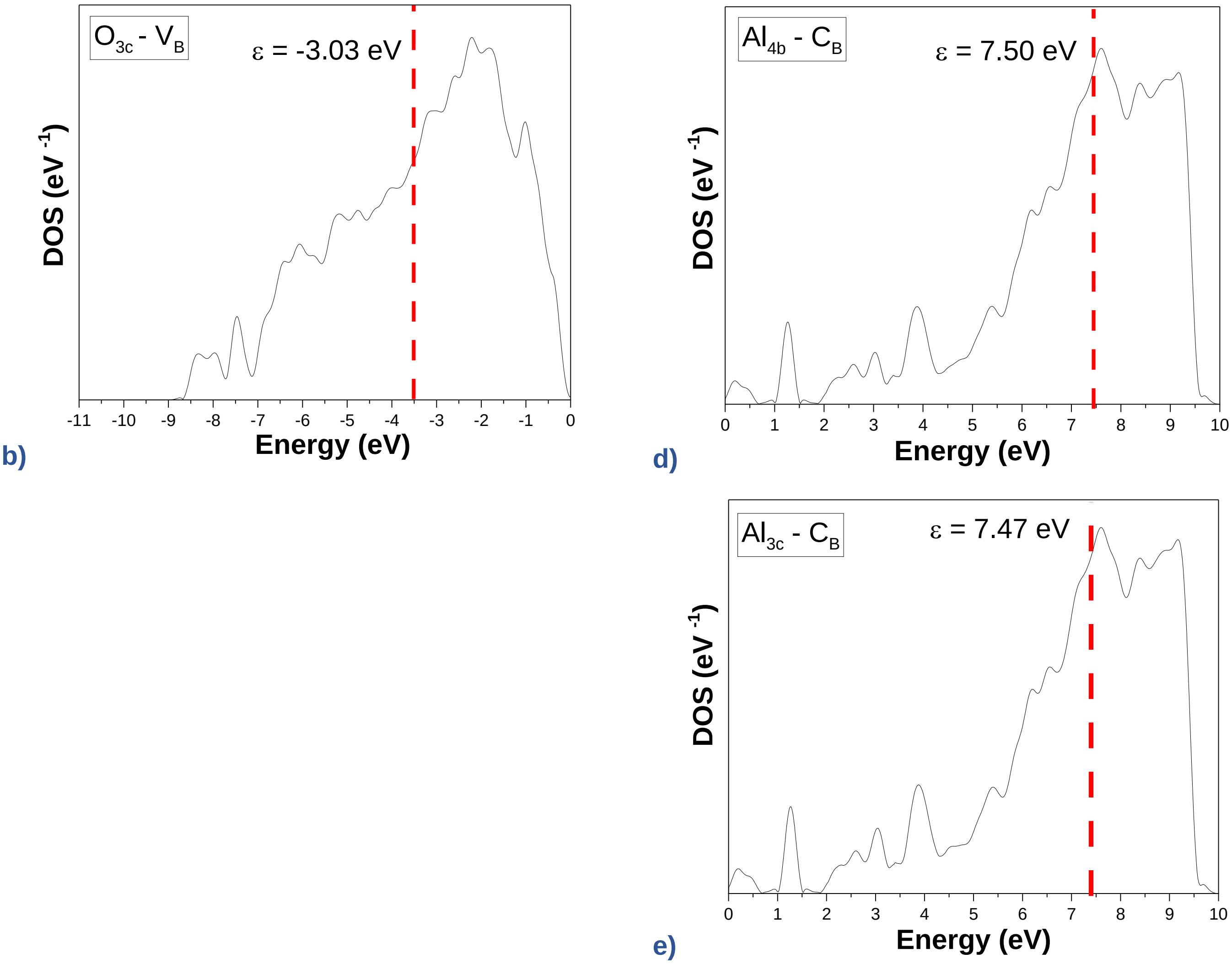

Figure S10. *PDOS for O-2p states for a)* O_{2c} *and b)* O_{3c}*; and PDOS for Al-3p states for c)* Al_{4a}*, d)* Al_{4b} *and* Al_{3c} *for hydroxylated γ-*Al_2O_3 *(110). Red dashed line represents the band centre in eV.*

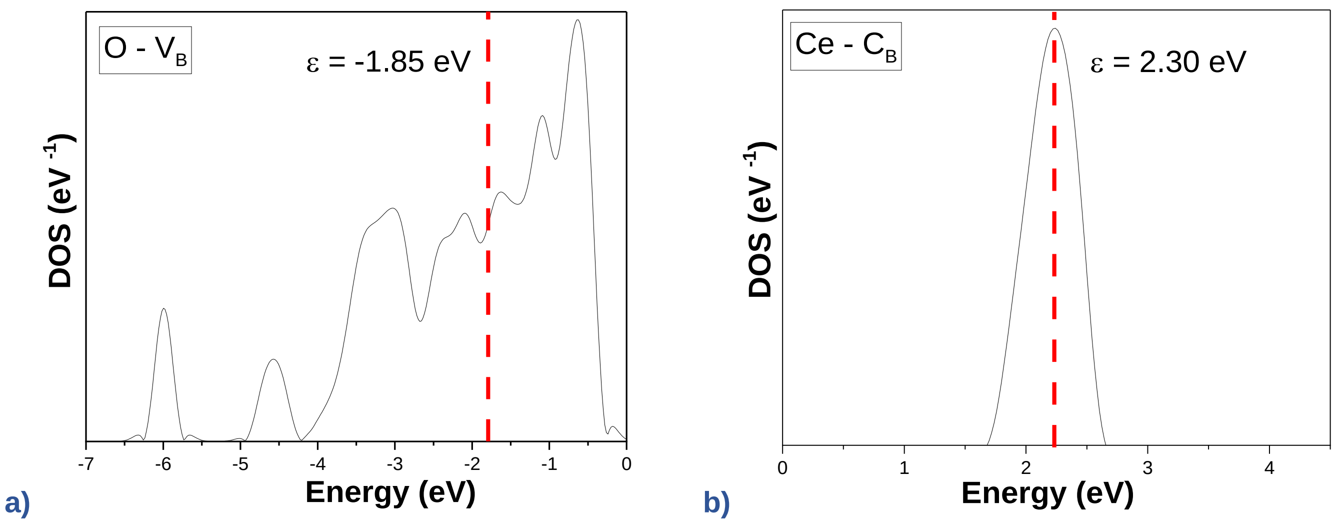

Figure S11. *PDOS for O-2p states for a) O; and PDOS for Ce 4f, 5d and 6s states for c) Ce for hydroxylated* CeO_2 *(111). Red dashed line represents the band centre in eV.*

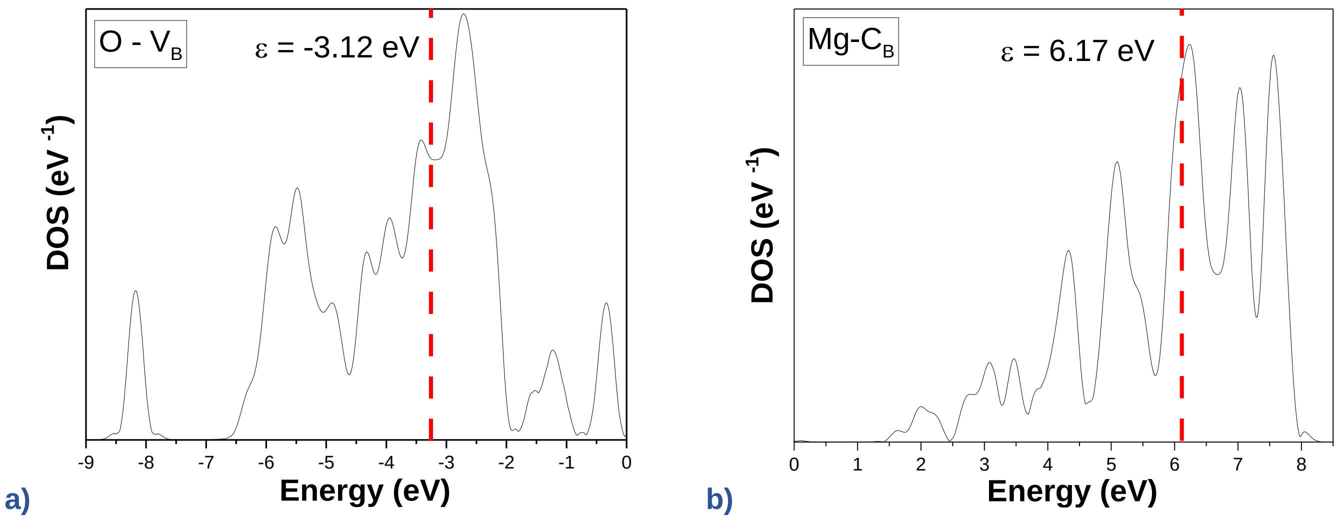

Figure S12. *PDOS for O-2p states for a) O; and PDOS for Mg-3s states for c) Mg for Hydroxylated MgO (100). Red dashed line represents the band centre in eV.*

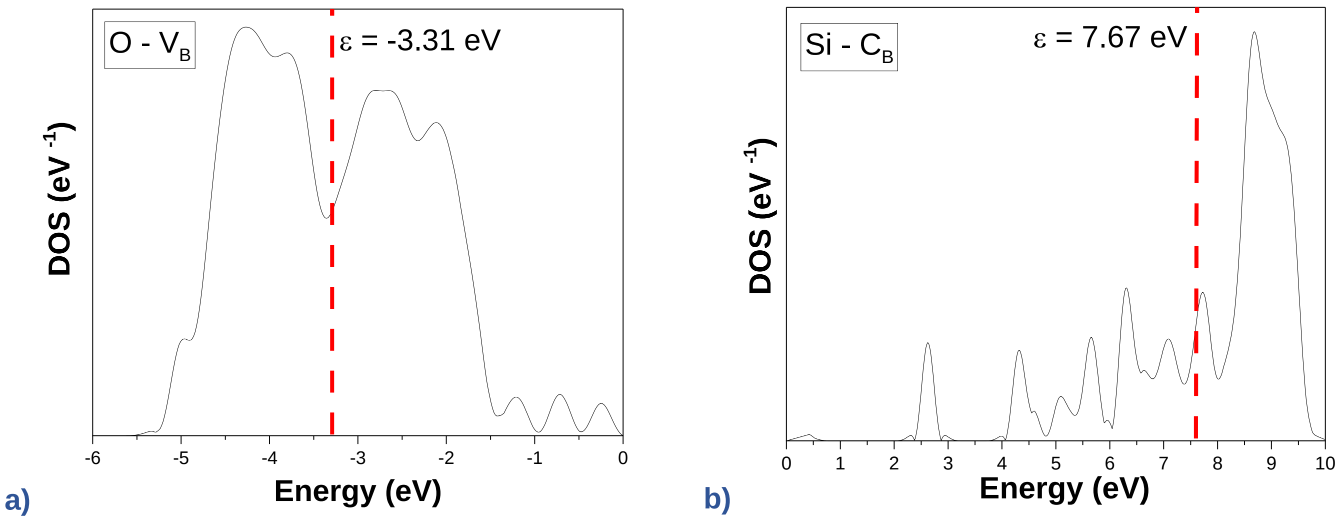

***Figure S13**. PDOS for O-2p states for a) O; and PDOS for Si-3p states for c) Si for Hydroxylated SiO_2 (100). Red dashed line represents the band centre in eV.*

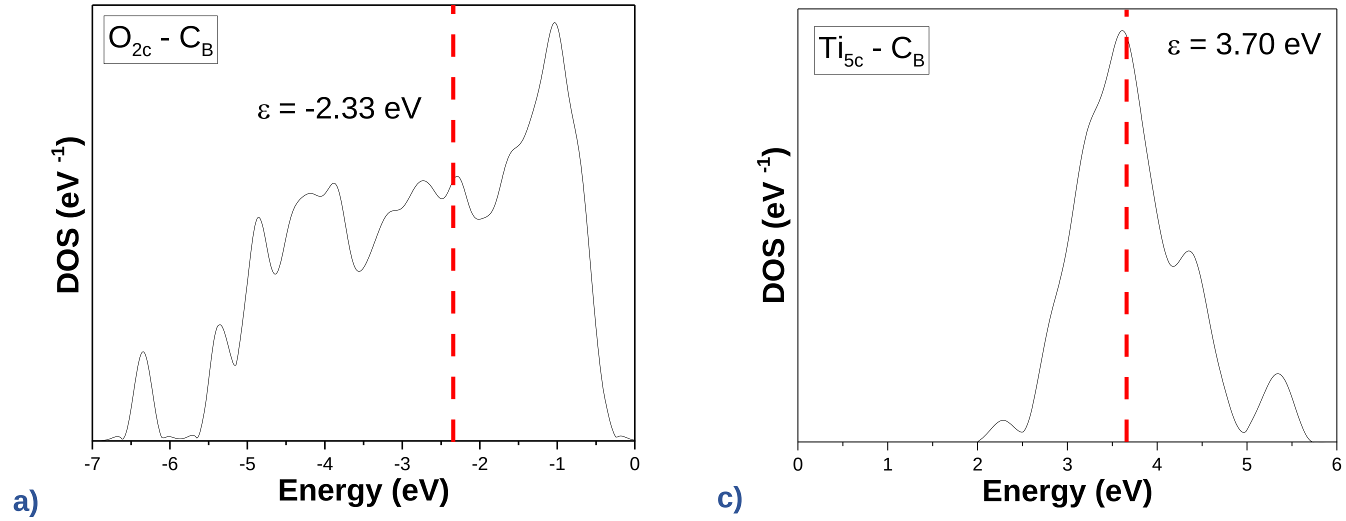

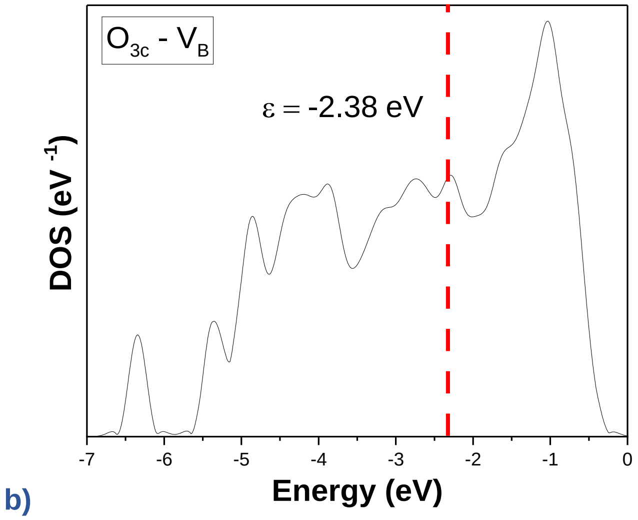

***Figure S14**. PDOS for O-2s and O-2p states for a) O_{2c} and b) O_{3c}; and PDOS for Ti 3d and 4p states for c) Ti for Hydroxylated a-TiO_2 (101). Red dashed line represents the band centre in eV.*

***Table S5**. Difference of Band centre (Δε) between clean and hydroxylated oxide surfaces for the unoccupied (V_B) and occupied regions (C_B).*

	Sites	Hydro ε_{CB} (eV)	Clean ε_{CB} (eV)	$\Delta\varepsilon_{CB}$ (eV)	Sites	Hydro ε_{VB} (eV)	Clean ε_{VB} (eV)	$\Delta\varepsilon_{VB}$ (eV)
**γ-Al_2O_3 (110)**	Al_{4a}	7.50	7.02	0.48	O_{2c}	−3.04	−3.43	0.39
	Al_{4b}	7.50	7.04	0.46	O_{3c}	−3.03	−3.45	0.42
	Al_{3c}	7.47	6.90	0.57				
**CeO_2 (111)**	Ce	2.30	2.23	0.07	O	−1.85	−1.69	−0.16
**MgO (100)**	Mg	6.17	7.20	−1.03	O	−3.12	−1.25	−1.87
**β-SiO_2 (100)**	Si	7.67	6.82	0.85	O	−3.31	−3.12	−0.19
**a-TiO_2 (101)**	Ti	3.70	3.50	0.20	O_{2c}	−2.33	−2.47	0.14
					O_{3c}	−2.38	−2.45	0.07

6. Adsorption on clean and hydroxylated surfaces (Illustrations)

Table S6. *Illustrations corresponding to the adsorption of a) guaiacol (GUA), b) catechol (CAT), c) phenol (PHE), d) anisole (ANI) and e) benzene (BEN) on clean γ-Al_2O_3 (110) at i) 90°, ii) 45° and iii) 0° geometries modes.*

	i) 90°	ii) 45°	iii) 0°
a) GUA	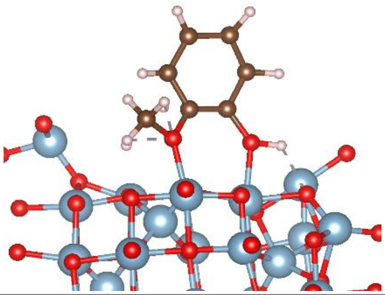	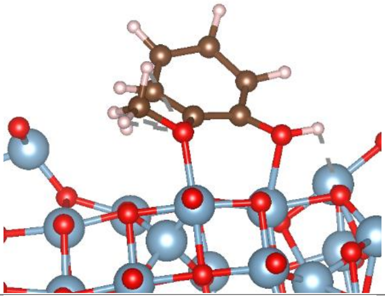	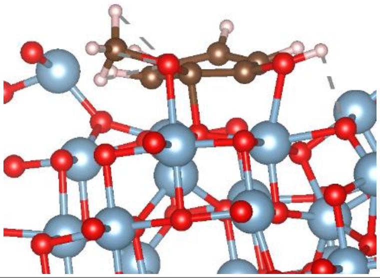
b) CAT	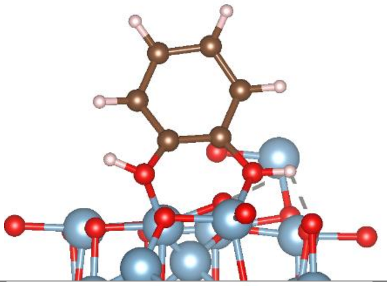	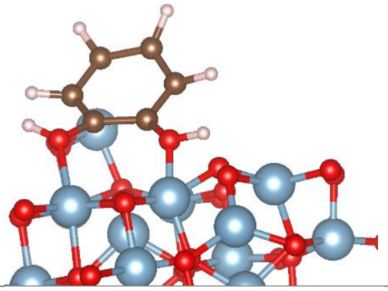	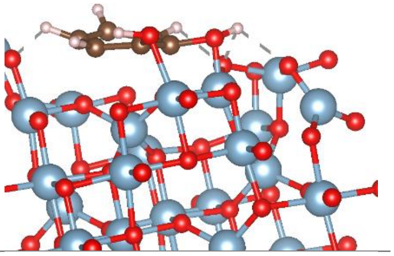
c) PHE	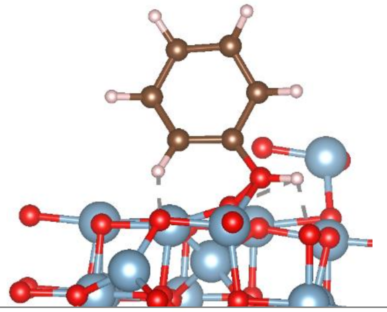	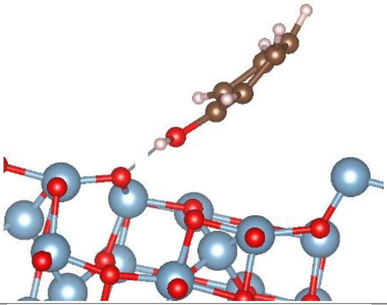	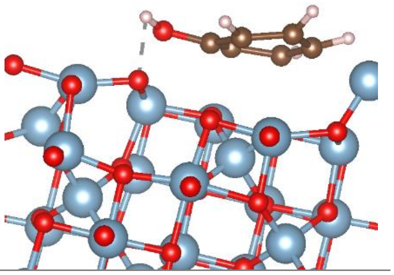
d) ANI	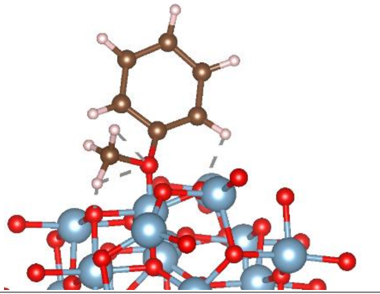	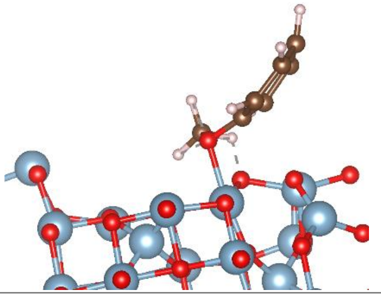	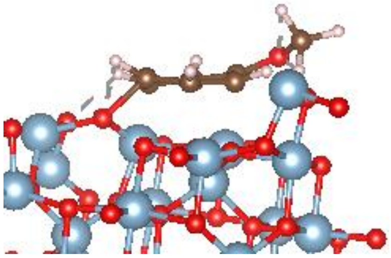

**e) BEN**	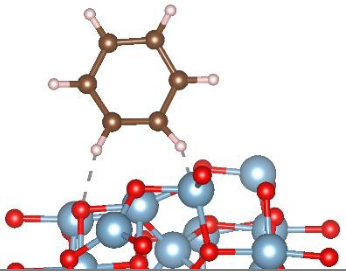	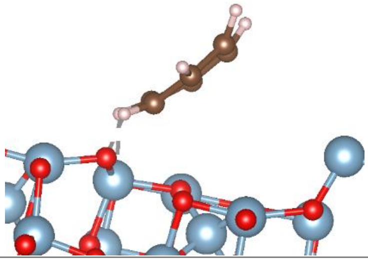	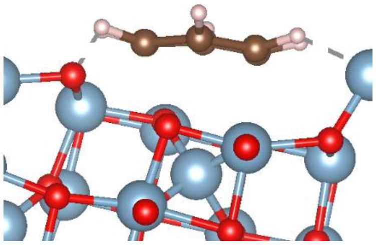

Table S7. *Illustrations corresponding to the adsorption of a) guaiacol (GUA), b) catechol (CAT), c) phenol (PHE), d) anisole (ANI) and e) benzene (BEN) on clean CeO_2 (111) at i) 90°, ii) 45° and iii) 0° geometries modes.*

	**i) 90°**	**ii) 45°**	**iii) 0°**
**a) GUA**	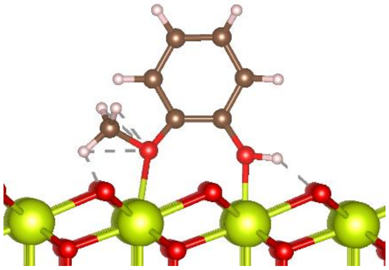	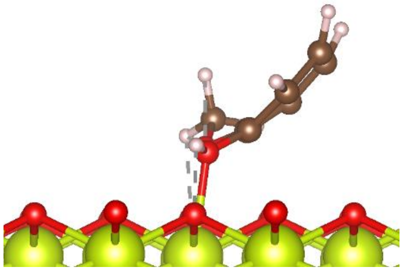	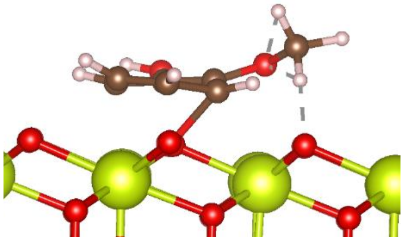
**b) CAT**	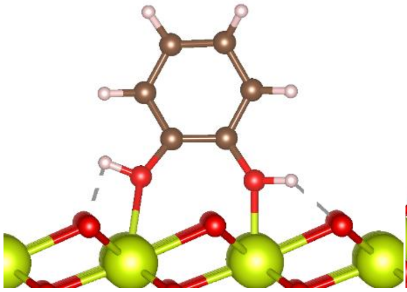	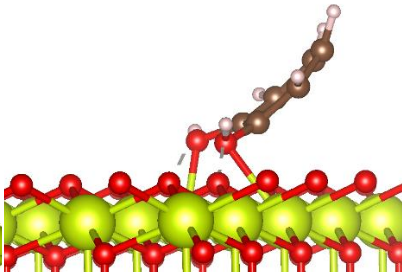	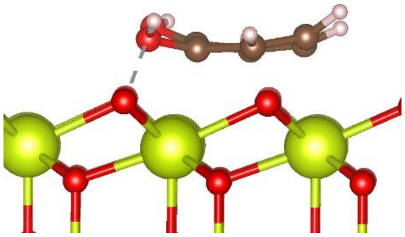
**c) PHE**	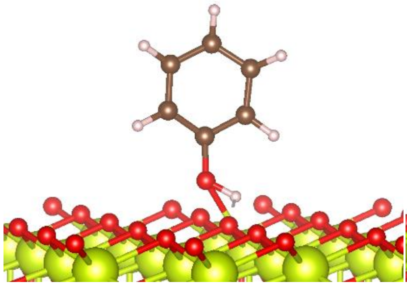	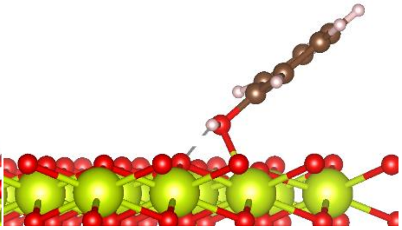	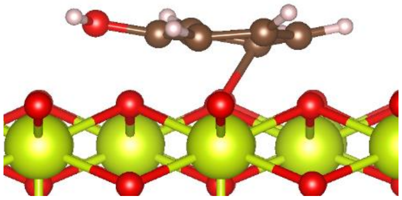

**d) ANI**	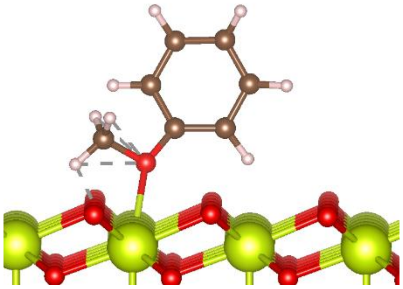	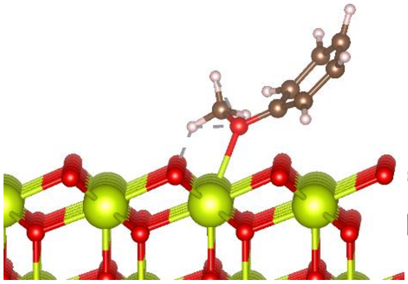	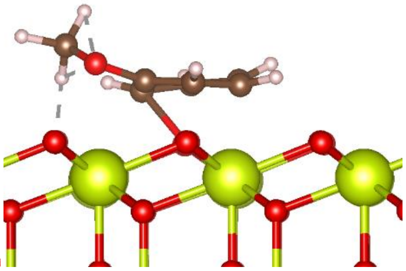
**e) BEN**	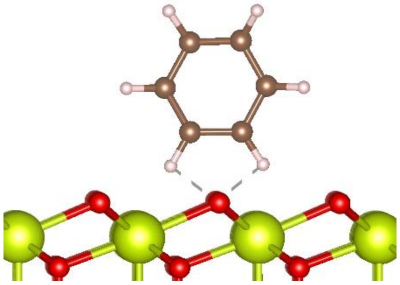	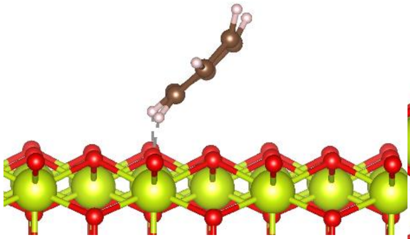	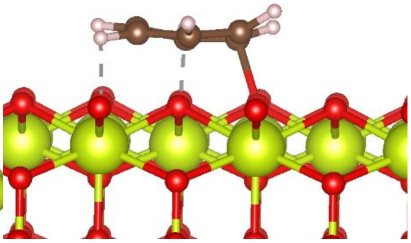

Table S8. *Illustrations corresponding to the adsorption of a) guaiacol (GUA), b) catechol (CAT), c) phenol (PHE), d) anisole (ANI) and e) benzene (BEN) on clean MgO (100) at i) 90°, ii) 45° and iii) 0° geometries modes.*

	**i) 90°**	**i) 45°**	**i) 0°**
**a) GUA**	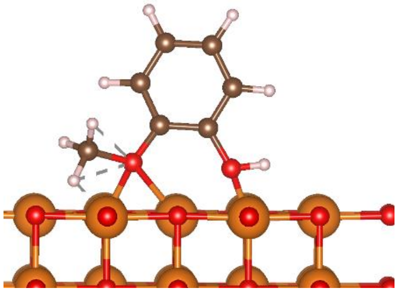	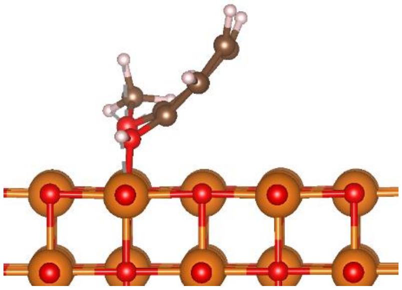	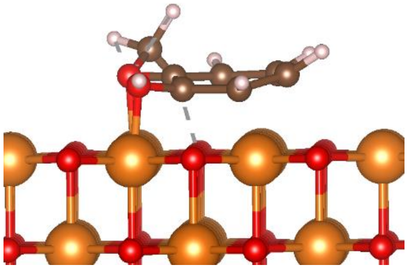
**b) CAT**	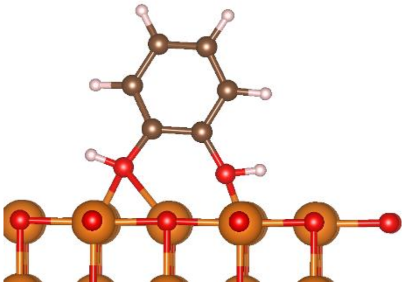	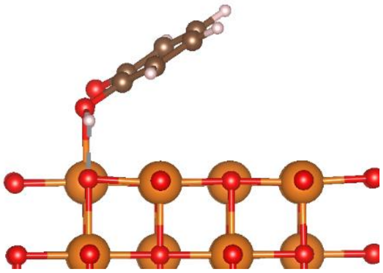	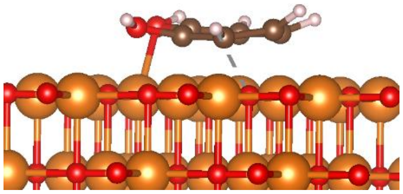

**c) PHE**	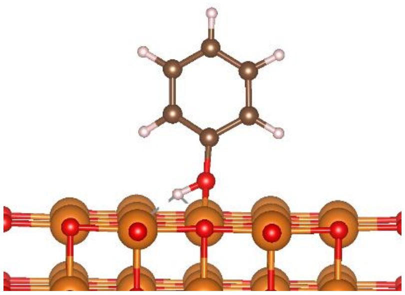	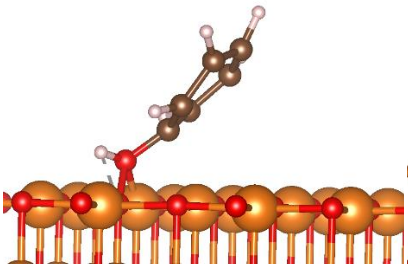	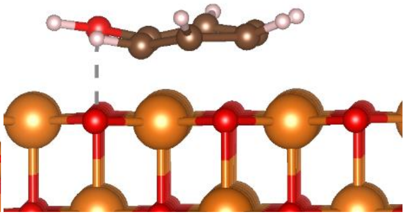
**d) ANI**	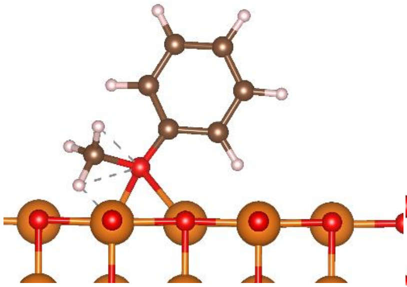	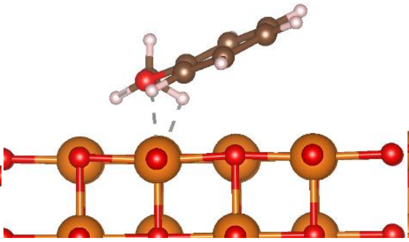	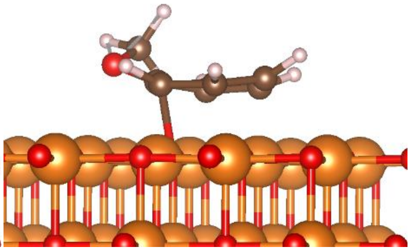
**e) BEN**	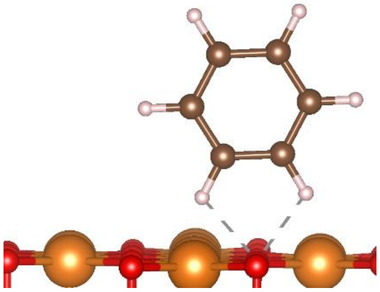	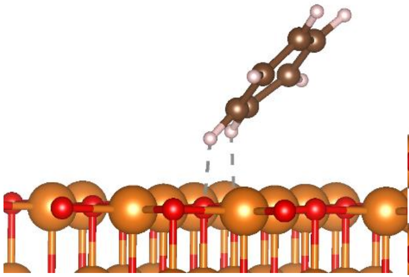	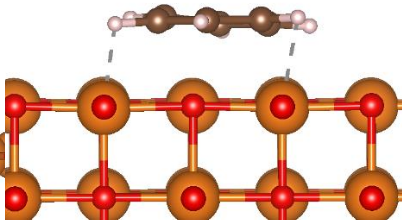

***Table S9**. Illustrations corresponding to the adsorption of a) guaiacol (GUA), b) catechol (CAT), c) phenol (PHE), d) anisole (ANI) and e) benzene (BEN) on clean a-TiO_2 (101) at i) 90°, ii) 45° and iii) 0° geometries modes.*

	**i) 90°**	**ii) 45°**	**iii) 0°**
**a) GUA**	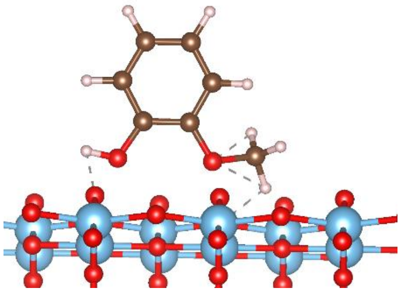	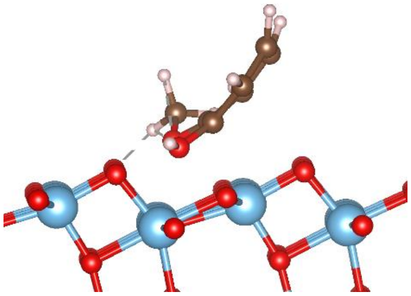	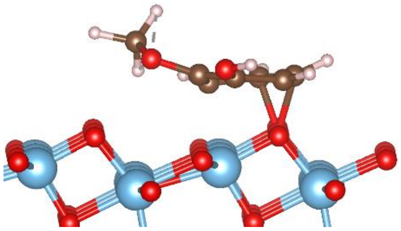

**b) CAT**	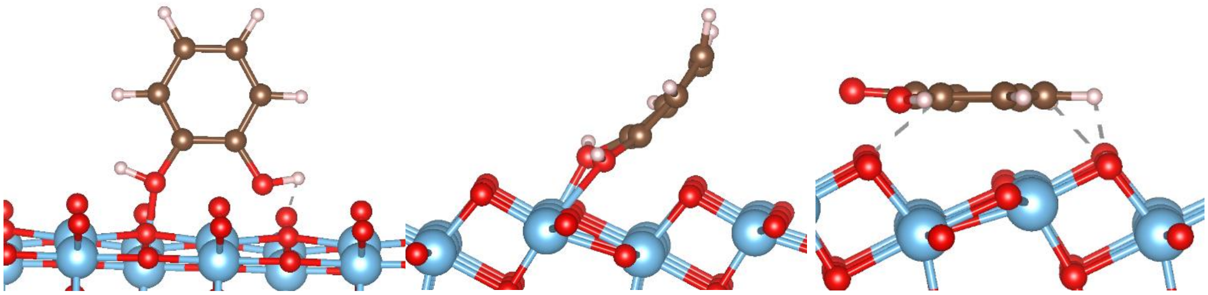
**c) PHE**	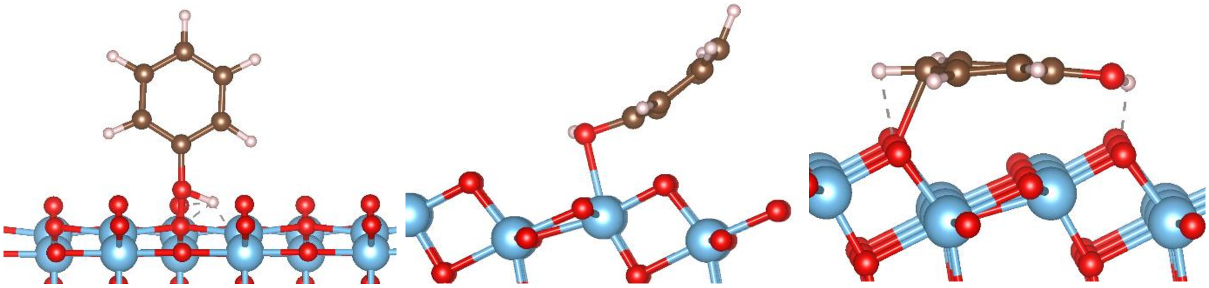
**d) ANI**	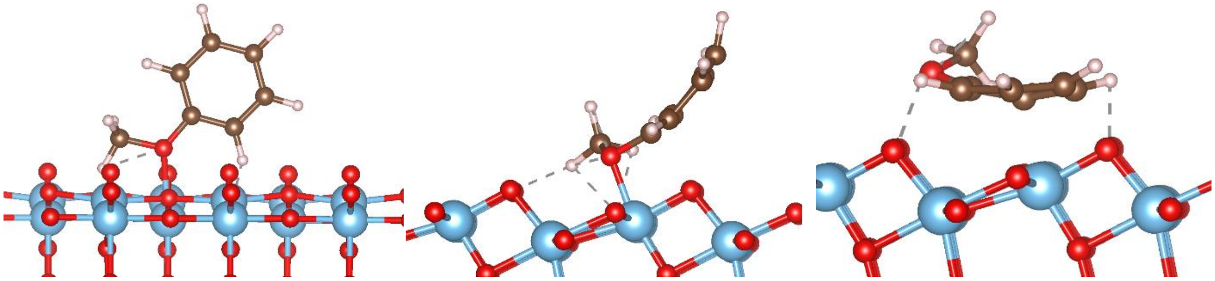
**e) BEN**	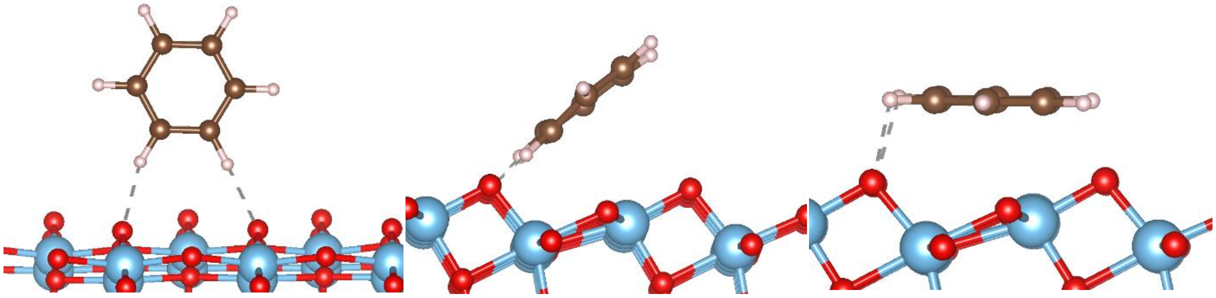

***Table S10**. Illustrations corresponding to the adsorption of a) guaiacol (GUA), b) catechol (CAT), c) phenol (PHE), d) anisole (ANI) and e) benzene (BEN) on hydroxylated CeO_2 (111) at i) 90°, ii) 45° and iii) 0° geometries modes.*

	**i) 90°**	**ii) 45°**	**iii) 0°**
**a) GUA**	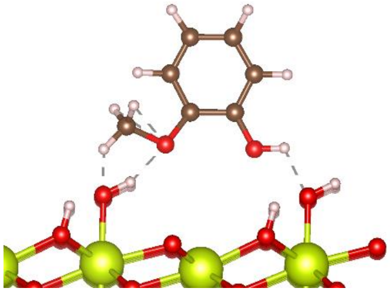	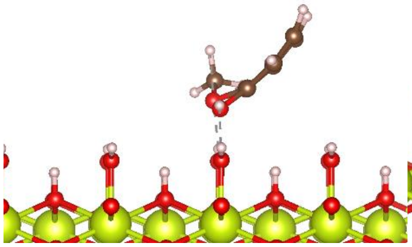	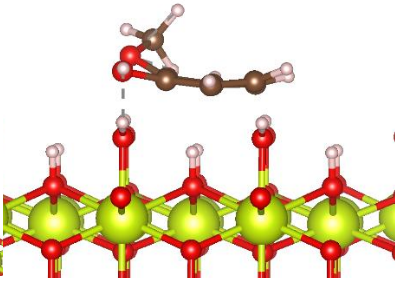
**b) CAT**	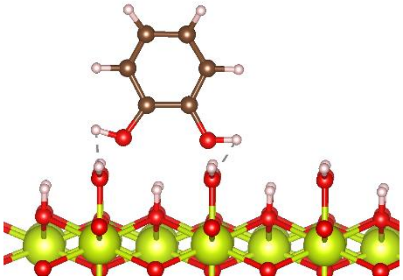	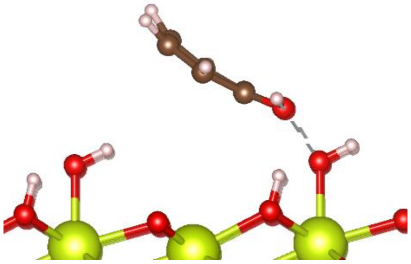	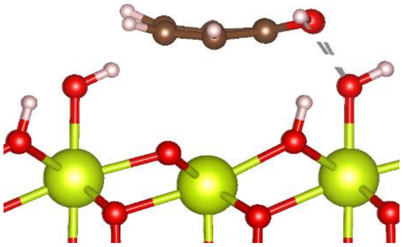
**c) PHE**	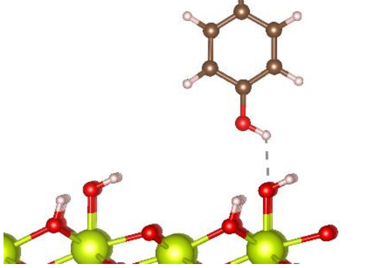	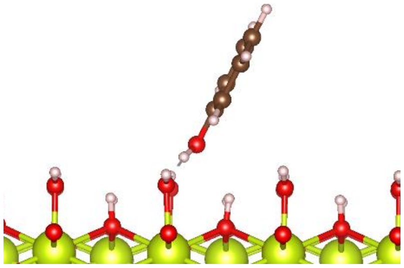	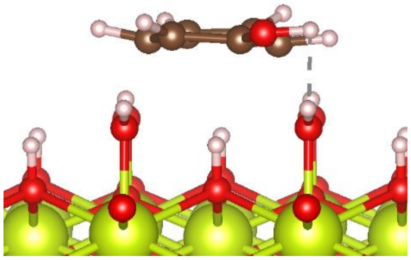
**d) ANI**	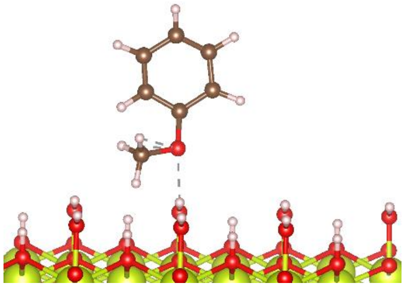	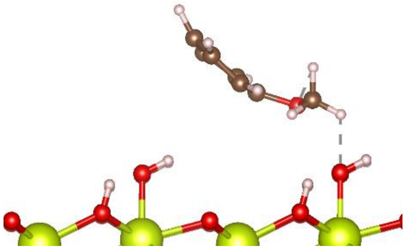	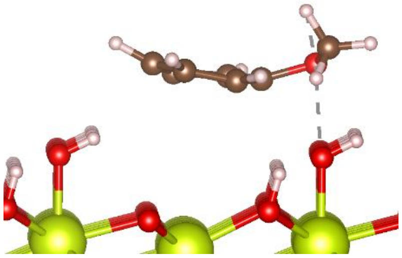

**e) BEN**	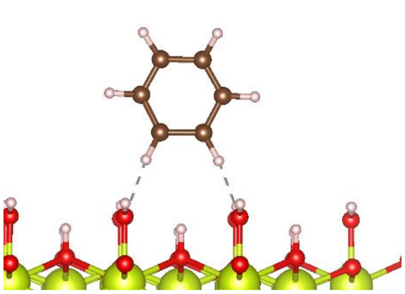	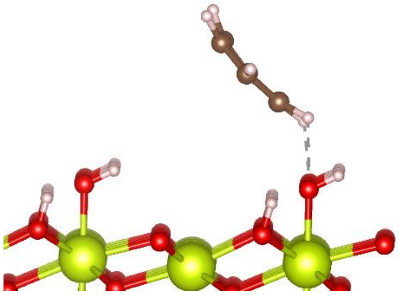	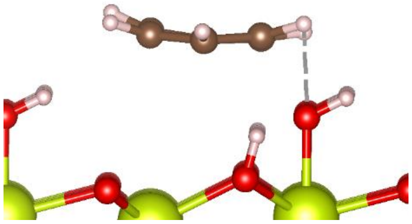

Table S11*. Illustrations corresponding to the adsorption of a) guaiacol (GUA), b) catechol (CAT), c) phenol (PHE), d) anisole (ANI) and e) benzene (BEN) on hydroxylated MgO (100) at i) 90°, ii) 45° and iii) 0° geometries modes.*

	**i) 90°**	**ii) 45°**	**iii) 0°**
**a) GUA**	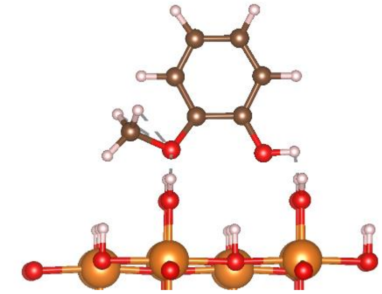	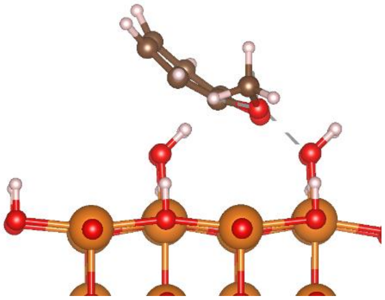	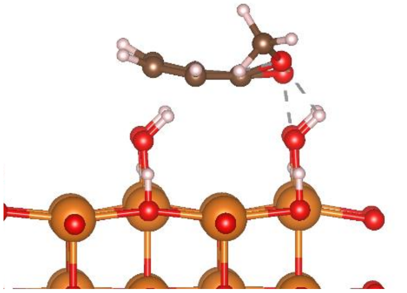
**b) CAT**	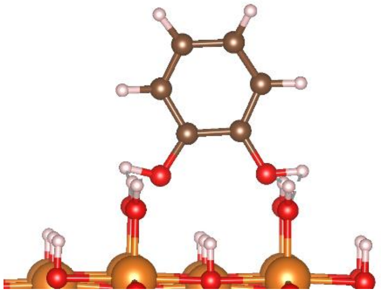	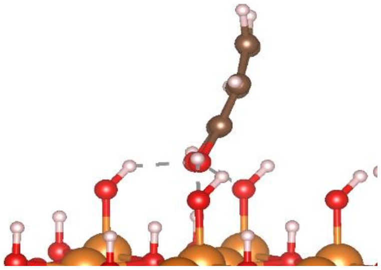	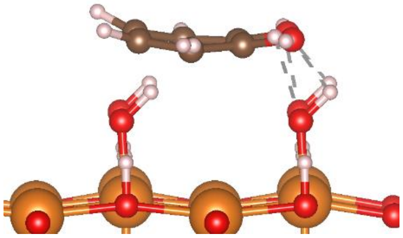
**c) PHE**	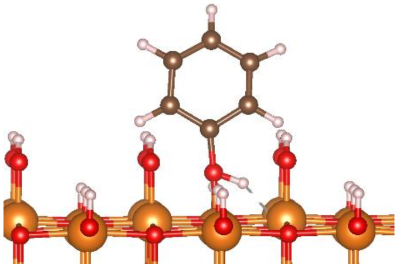	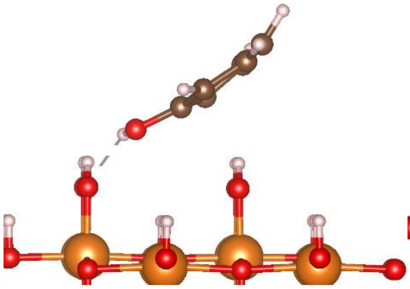	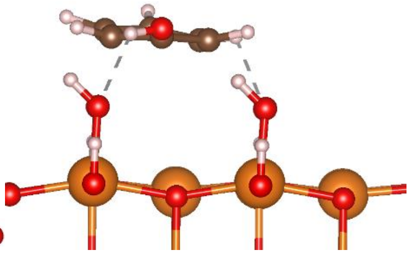

**d) ANI**	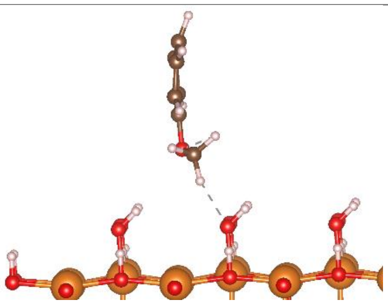	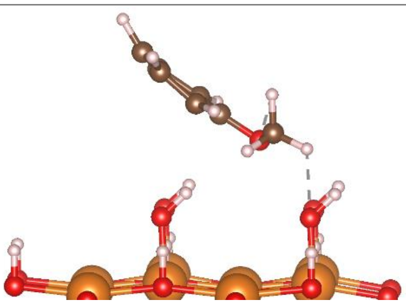	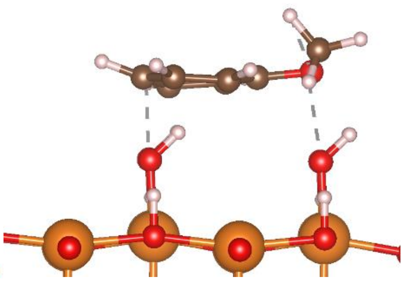
**e) BEN**	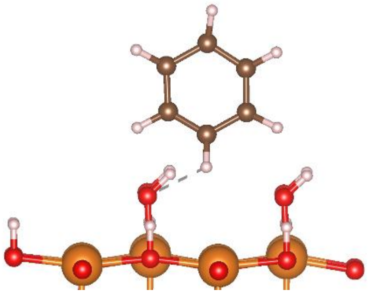	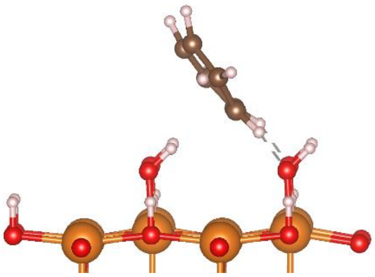	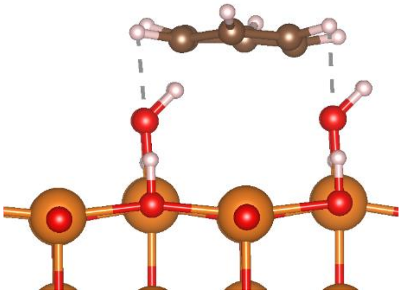

Table S12. *Illustrations corresponding to the adsorption of a) guaiacol (GUA), b) catechol (CAT), c) phenol (PHE), d) anisole (ANI) and e) benzene (BEN) on hydroxylated β-SiO$_2$ (100) at i) 90°, ii) 45° and iii) 0° geometries modes.*

	**i) 90°**	**ii) 45°**	**iii) 0°**
**a) GUA**	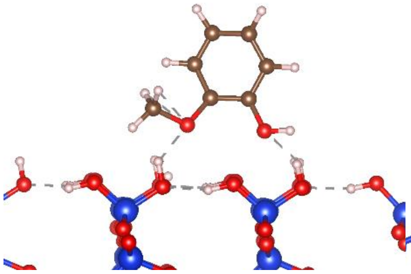	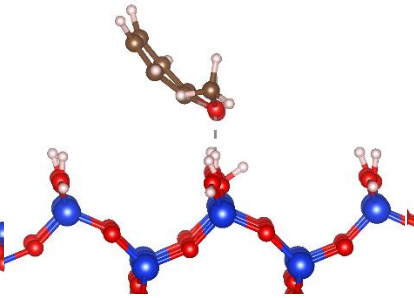	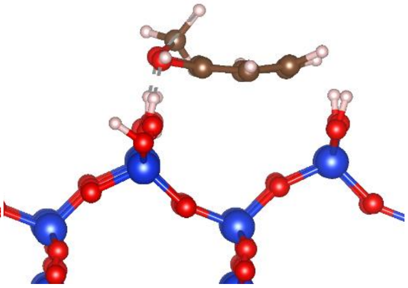
**b) CAT**	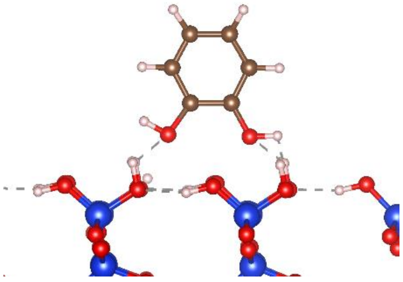	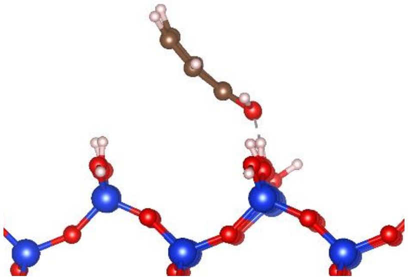	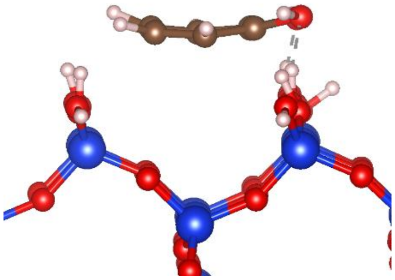

**c) PHE**	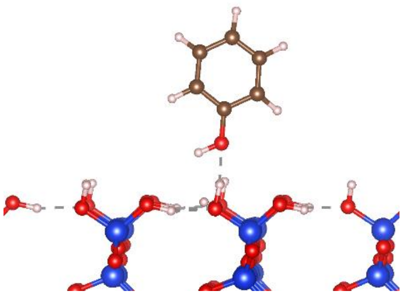	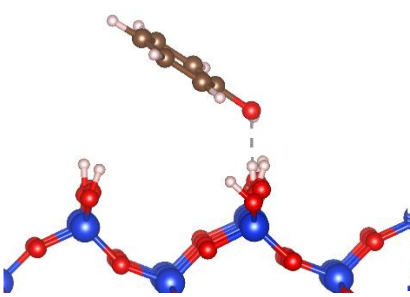	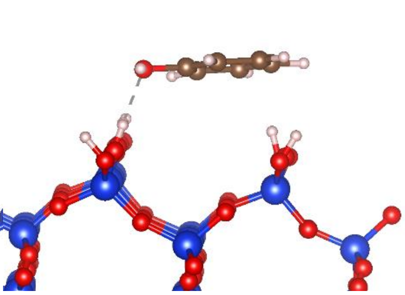
**d) ANI**	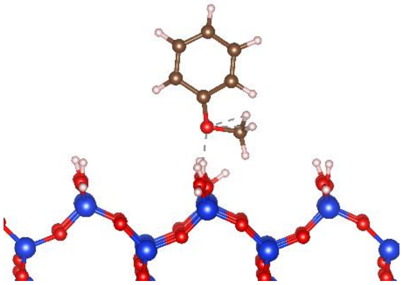	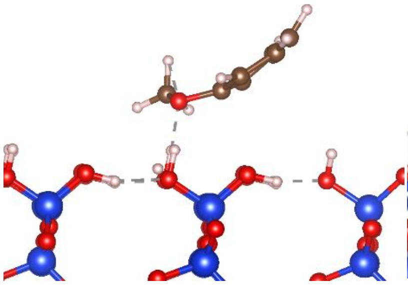	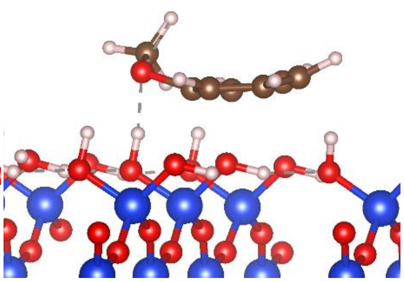
**e) BEN**	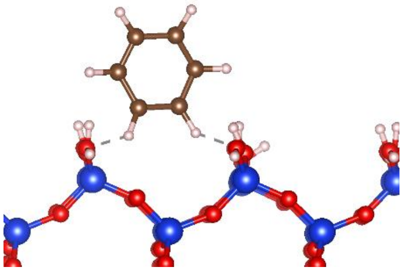	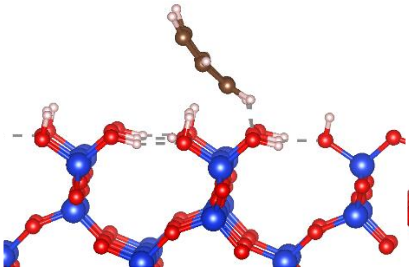	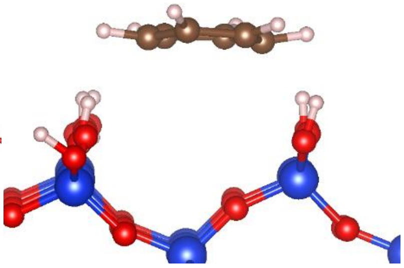

Table S13*. Illustrations corresponding to the adsorption of a) guaiacol (GUA), b) catechol (CAT), c) phenol (PHE), d) anisole (ANI) and e) benzene (BEN) on hydroxylated a-TiO$_2$ (101) at i) 90°, ii) 45° and iii) 0° geometries modes.*

	**i) 90°**	**ii) 45°**	**iii) 0°**
**a) GUA**	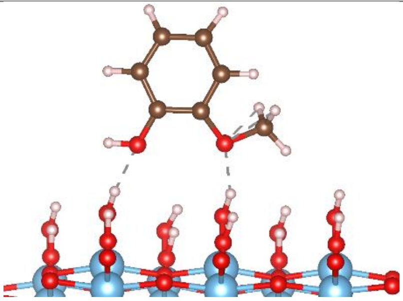	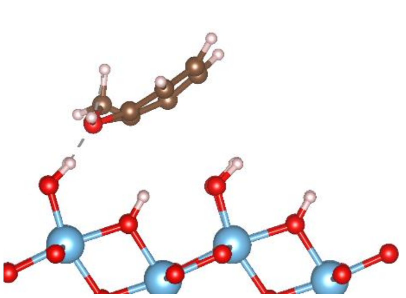	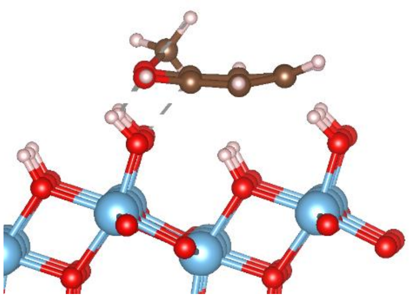

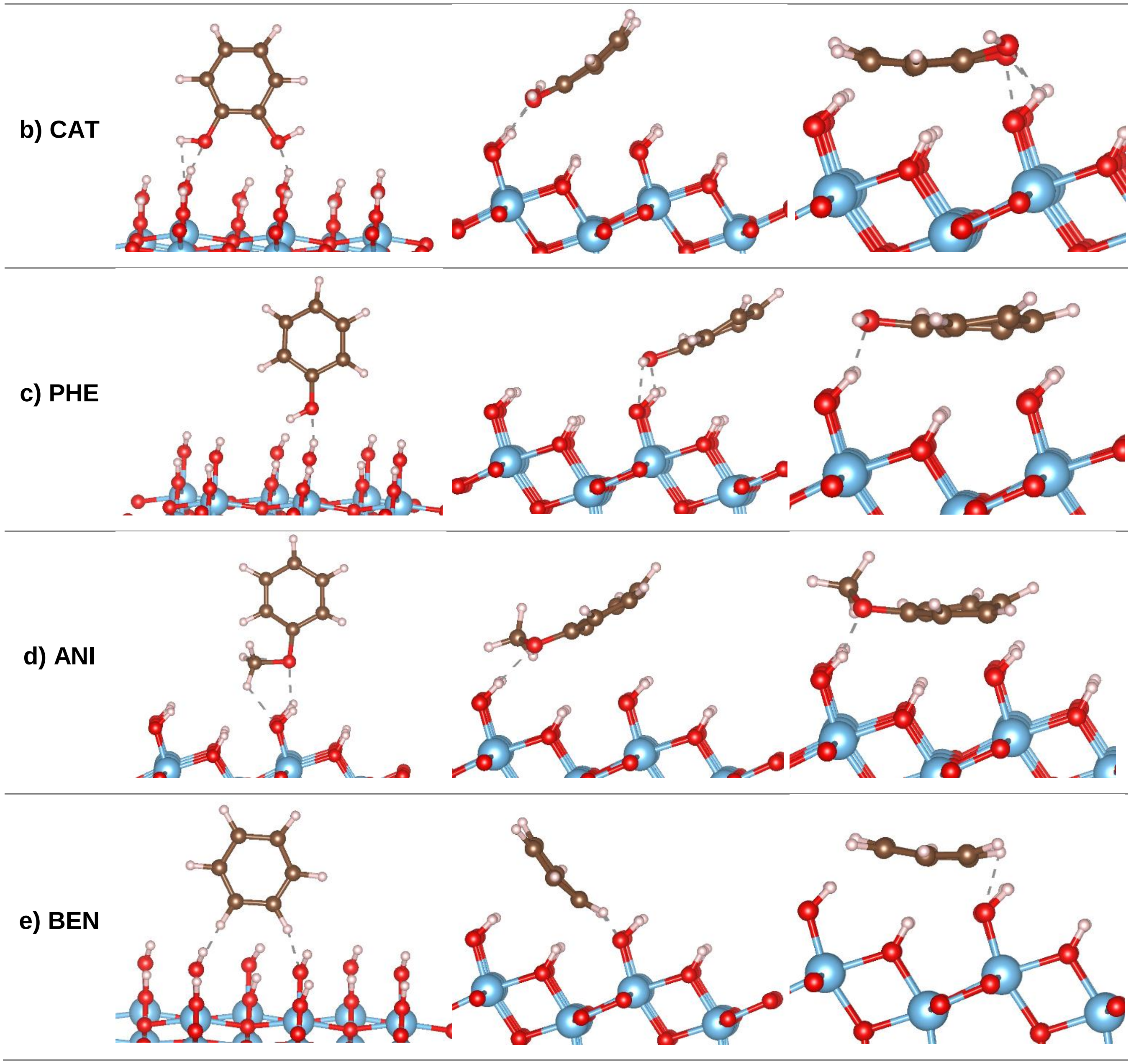
b) CAT
c) PHE
d) ANI
e) BEN

7. Model compounds adsorption on clean surfaces

Summarised of model compounds such as a) guaiacol (GUA), b) catechol (CAT), c) phenol (PHE), d) anisole (ANI) and e) benzene (BEN) adsorption energy on clean oxide surfaces is shown in ***Table S14-S17***. Bader analysis charge were carried out to get an insight of the nature of the bonding between the compound and the surface in |e|. Moreover, bond distances between oxygen from the molecule (d-O_1 = hydroxyl group and d-O_2 = methoxy group) were calculated on different geometries modes (90°, 45° and 0°).

Table S14. *Calculated adsorption energies (E_{ads}), distance oxygen-surface (d) and Bader charge (q) for the clean γ-Al_2O_3 (110) surface in different geometries modes.*

			**γ-Al_2O_3 (110) - clean**	
		**90°**	**45°**	**0°**
**GUA**	**E_{ads} (eV)**	−1.14	−1.78	−1.47
	**d-O_1 (−OH) (Å)**	2.05	1.93	1.91
	**d-O_2 (−OCH_3) (Å)**	2.14	2.10	2.24
	**q |e^-|**	0.03	0.17	0.80
**CAT**	**E_{ads} (eV)**	−1.71	−0.71	−1.75
	**d-O_{1A} (−OH) (Å)**	2.03	1.85	2.04
	**d-O_{1B} (−OH) (Å)**	1.75	1.79	1.68
	**q |e^-|**	0.01	0.16	0.47
**PHE**	**E_{ads} (eV)**	−0.85	−0.91	−1.19
	**d-O_1 (−OH) (Å)**	2.18	1.71	1.68
	**q |e^-|**	0.14	0.48	0.49
**ANI**	**E_{ads} (eV)**	−0.99	−1.05	−1.56
	**d-O_2 (−OCH_3) (Å)**	2.16	2.08	2.07
	**q |e^-|**	0.55	0.14	0.34
**BEN**	**E_{ads} (eV)**	−0.96	−1.45	−1.33
	**q |e^-|**	0.07	0.06	0.24

Table S15. *Calculated adsorption energies (E_{ads}), distance oxygen-surface (d) and Bader charge (q) for the clean CeO_2 (111) surface in different geometries modes.*

	CeO_2 (111) - clean			
		**90°**	**45°**	**0°**
**GUA**	**E_{ads} (eV)**	−0.67	−0.91	−0.73
	**d-O_1 (−OH) (Å)**	2.02	1.89	1.77
	**d-O_2 (−OCH_3) (Å)**	2.10	1.91	2.05
	**q |e^-|**	0.03	0.02	0.23
**CAT**	**E_{ads} (eV)**	−0.77	−0.92	−0.76
	**d-O_{1A} (−OH) (Å)**	1.82	2.02	1.88
	**d-O_{1B} (−OH) (Å)**	2.10	2.21	1.85
	**q |e^-|**	0.06	0.31	0.26
**PHE**	**E_{ads} (eV)**	−0.38	−0.79	−0.61
	**d-O_1 (−OH) (Å)**	1.93	1.79	1.80
	**q |e^-|**	0.02	0.02	0.14
**ANI**	**E_{ads} (eV)**	−0.48	−0.69	−0.60
	**d-O_2 (−OCH_3) (Å)**	1.92	1.62	1.64
	**q |e^-|**	0.04	0.03	0.47
**BEN**	**E_{ads} (eV)**	−0.45	−0.55	0.42
	**q |e^-|**	0.02	0.02	0.45

Table S16. *Calculated adsorption energies (E_{ads}), distance oxygen-surface (d) and Bader charge (q in |e|) for the clean MgO (100) surface in different geometries modes.*

	MgO (100) - clean			
		**90°**	**45°**	**0°**
**GUA**	**E_{ads} (eV)**	−0.79	−1.09	−1.14
	**d-O_1 (−OH) (Å)**	1.89	1.58	1.74
	**d-O_2 (−OCH_3) (Å)**	1.95	2.13	1.98
	**q |e^-|**	0.36	0.72	1.32
**CAT**	**E_{ads} (eV)**	−0.31	−0.99	−1.12
	**d-O_{1A} (−OH) (Å)**	1.91	1.87	1.76
	**d-O_{1B} (−OH) (Å)**	1.99	1.67	1.72

	q |e⁻|	0.53	0.30	0.45
PHE	E_{ads} (eV)	−0.63	−0.73	−0.82
	d-O_1 (−OH) (Å)	1.91	1.71	1.60
	q |e⁻|	0.10	0.47	0.16
ANI	E_{ads} (eV)	−0.43	−0.67	−0.85
	d-O_2 (−OCH_3) (Å)	1.99	2.04	2.05
	q |e⁻|	0.04	0.47	0.24
BEN	E_{ads} (eV)	−0.54	−0.45	−0.64
	q |e⁻|	0.02	0.06	0.12

Table S17. *Calculated adsorption energies (E_{ads}), distance oxygen-surface (d) and Bader charge (q) for the clean a-TiO_2 (101) surface in different geometries modes.*

a-TiO_2 (101) - clean				
		**90°**	**45°**	**0°**
**GUA**	**E_{ads} (eV)**	−0.71	−0.85	−0.73
	**d-O_1 (−OH) (Å)**	2.11	2.18	2.25
	**d-O_2 (−OCH_3) (Å)**	2.22	2.31	2.33
	**q |e⁻|**	0.71	0.61	0.84
**CAT**	**E_{ads} (eV)**	−0.80	−0.67	−0.59
	**d-O_{1A} (−OH) (Å)**	2.03	2.13	2.33
	**d-O_{1B} (−OH) (Å)**	1.86	2.04	2.32
	**q |e⁻|**	0.16	0.18	0.40
**PHE**	**E_{ads} (eV)**	−0.55	−0.71	−0.64
	**d-O_1 (−OH) (Å)**	1.85	2.03	2.07
	**q |e⁻|**	0.29	0.53	0.30
**ANI**	**E_{ads} (eV)**	−0.29	−0.63	−0.57
	**d-O_2 (−OCH_3) (Å)**	2.06	2.23	2.19
	**q |e⁻|**	0.24	0.42	0.90
**BEN**	**E_{ads} (eV)**	−0.24	−0.58	−0.49
	**q |e⁻|**	0.94	0.02	0.46

8. Model compounds adsorption on hydroxylated surfaces

Summarised of model compounds such as a) guaiacol (GUA), b) catechol (CAT), c) phenol (PHE), d) anisole (ANI) and e) benzene (BEN) adsorption energy on hydroxylated (HYD) oxide surfaces is shown in ***Table S18-S22***. Bader analysis charge were carried out to get an insight of the nature of the bonding between the compound and the surface in $|e^-|$. Moreover, bond distances between oxygen from the molecule (d-O_1 = hydroxyl group and d-O_2 = methoxy group) were calculated on different geometries modes (90°, 45° and 0°).

Table S18. *Calculated adsorption energies (E_{ads}), distance oxygen-surface (d) and Bader charge (q) for the hydroxylated γ-Al_2O_3 (110) surface in different geometries modes.*

		**γ-Al_2O_3 (110) - HYD**		
		**90°**	**45°**	**0°**
**GUA**	**E_{ads} (eV)**	−1.50	−1.16	−1.91
	**d-O_1 (−OH) (Å)**	2.68	2.74	2.51
	**d-O_2 (−OCH_3) (Å)**	2.53	2.69	2.30
	**q $|e^-|$**	0.20	0.87	0.92
**CAT**	**E_{ads} (eV)**	−1.59	−1.89	−1.50
	**d-O_{1A} (−OH) (Å)**	1.54	1.78	1.99
	**d-O_{1B} (−OH) (Å)**	2.07	1.89	2.03
	**q $|e^-|$**	−0.45	0.30	0.26
**PHE**	**E_{ads} (eV)**	−0.71	−1.02	−1.26
	**d-O_1 (−OH) (Å)**	2.12	1.74	2.04
	**q $|e^-|$**	0.06	0.31	0.32
**ANI**	**E_{ads} (eV)**	−1.42	−1.48	−1.69
	**d-O_2 (−OCH_3) (Å)**	2.08	2.01	2.02
	**q $|e^-|$**	0.07	0.04	0.27
**BEN**	**E_{ads} (eV)**	−1.19	−1.61	−1.53
	**q $|e^-|$**	0.04	0.39	0.98

Table S19. *Calculated adsorption energies (E_{ads}), distance oxygen-surface (d) and Bader charge (q) for the hydroxylated CeO_2 (111) surface in different geometries modes.*

		CeO_2 (111) - HYD		
		**90°**	**45°**	**0°**
**GUA**	**E_{ads} (eV)**	−1.03	−1.26	−1.23
	**d-O_1 (−OH) (Å)**	1.65	1.60	1.65
	**d-O_2 (−OCH_3) (Å)**	2.17	2.30	2.09
	**q |e^-|**	0.25	0.60	0.64
**CAT**	**E_{ads} (eV)**	−1.00	−1.38	−1.32
	**d-O_{1A} (−OH) (Å)**	1.59	1.67	1.63
	**d-O_{1B} (−OH) (Å)**	1.60	1.65	1.60
	**q |e^-|**	0.31	0.31	0.40
**PHE**	**E_{ads} (eV)**	−0.65	−0.72	−0.98
	**d-O_1 (−OH) (Å)**	1.66	1.62	1.52
	**q |e^-|**	0.39	0.65	0.48
**ANI**	**E_{ads} (eV)**	−0.25	−0.42	−0.74
	**d-O_2 (−OCH_3) (Å)**	2.26	2.22	2.28
	**q |e^-|**	0.05	0.01	0.08
**BEN**	**E_{ads} (eV)**	−0.29	−0.35	−0.61
	**q |e^-|**	0.03	0.03	0.04

Table S20. *Calculated adsorption energies (E_{ads}), distance oxygen-surface (d) and Bader charge (q) for the hydroxylated MgO (100) surface in different geometries modes.*

		MgO (100)-HYD		
		**90°**	**45°**	**0°**
**GUA**	**E_{ads} (eV)**	−0.37	−0.49	−0.69
	**d-O_1 (−OH) (Å)**	1.61	1.68	1.57
	**d-O_2 (−OCH_3) (Å)**	2.15	2.27	2.38
	**q |e^-|**	0.70	0.50	0.60
**CAT**	**E_{ads} (eV)**	−0.54	−0.61	−0.28
	**d-O_{1A} (−OH) (Å)**	1.54	1.63	1.72
	**d-O_{1B} (−OH) (Å)**	1.55	1.51	1.71

	**q |e⁻|**	0.11	0.69	0.38
**PHE**	**E_{ads} (eV)**	−0.66	−0.56	−0.75
	**d-O_1 (−OH) (Å)**	1.64	1.56	1.51
	**q |e^-|**	0.10	0.10	0.27
**ANI**	**E_{ads} (eV)**	−0.24	−0.31	−0.57
	**d-O_2 (−OCH_3) (Å)**	2.24	2.31	2.24
	**q |e^-|**	0.10	0.15	0.14
**BEN**	**E_{ads} (eV)**	−0.26	−0.35	−0.50
	**q |e^-|**	0.03	0.02	0.06

Table S21. *Calculated adsorption energies (E_{ads}), distance oxygen-surface (d) and Bader charge (q) for the hydroxylated β-SiO_2 (100) surface in different geometries modes.*

		**β-SiO_2 (100) - HYD**		
		**90°**	**45°**	**0°**
**GUA**	**E_{ads} (eV)**	−0.26	−0.44	−0.51
	**d-O_1 (−OH) (Å)**	2.51	2.22	2.21
	**d-O_2 (−OCH_3) (Å)**	2.53	2.26	2.22
	**q |e^-|**	0.06	0.20	0.54
**CAT**	**E_{ads} (eV)**	−0.21	−0.31	−0.42
	**d-O_{1A} (−OH) (Å)**	2.26	2.26	2.26
	**d-O_{1B} (−OH) (Å)**	2.24	2.19	2.11
	**q |e^-|**	0.07	0.04	0.57
**PHE**	**E_{ads} (eV)**	−0.39	−0.30	−0.65
	**d-O_1 (−OH) (Å)**	1.92	1.80	1.87
	**q |e^-|**	0.01	0.21	0.40
**ANI**	**E_{ads} (eV)**	−0.36	−0.52	−0.54
	**d-O_2 (−OCH_3) (Å)**	1.87	1.87	1.94
	**q |e^-|**	0.14	0.23	0.58
**BEN**	**E_{ads} (eV)**	−0.10	−0.41	−0.50
	**q |e^-|**	0.18	0.49	0.84

Table S21. *Calculated adsorption energies (E_{ads}), distance oxygen-surface (d) and Bader charge (q) for the hydroxylated a-TiO_2 (101) surface in different geometries modes.*

a-TiO_2 (101) - HYD				
		**90°**	**45°**	**0°**
**GUA**	**E_{ads} (eV)**	−1.36	−1.62	−1.26
	**d-O_1 (−OH) (Å)**	2.17	2.12	1.87
	**d-O_2 (−OCH_3) (Å)**	1.95	1.95	2.22
	**q |e^-|**	0.11	0.64	0.55
**CAT**	**E_{ads} (eV)**	−0.78	−1.59	−0.93
	**d-O_{1A} (−OH) (Å)**	2.07	1.63	2.20
	**d-O_{1B} (−OH) (Å)**	2.28	1.97	2.22
	**q |e^-|**	0.09	0.15	0.73
**PHE**	**E_{ads} (eV)**	−0.94	−0.73	−1.05
	**d-O_1 (−OH) (Å)**	2.10	2.03	1.93
	**q |e^-|**	0.16	0.56	0.63
**ANI**	**E_{ads} (eV)**	−0.96	−1.23	−1.18
	**d-O_2 (−OCH_3) (Å)**	2.19	2.18	1.83
	**q |e^-|**	0.40	0.40	0.65
**BEN**	**E_{ads} (eV)**	−0.89	−0.96	−0.75
	**q |e^-|**	0.15	0.32	0.13

9. Adsorption energies for the model compounds versus band centres

Figure S15 displays a volcano type plot where ε_{VB} represents the Lewis basicity strength (from weak to strong strength: from left to right). In contrast, ε_{CB} represents the Lewis acid strength (from weak to strong strength: from left to right).

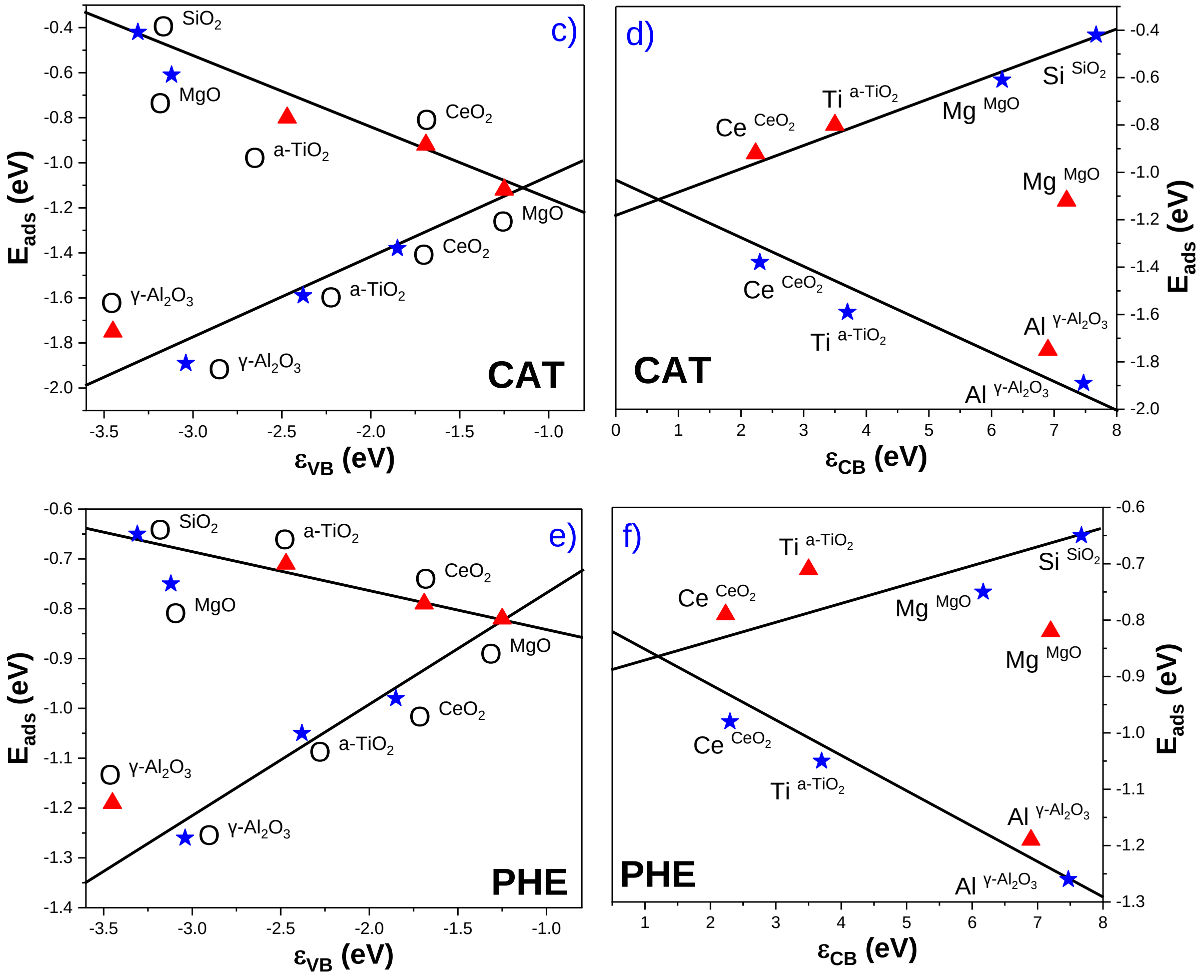

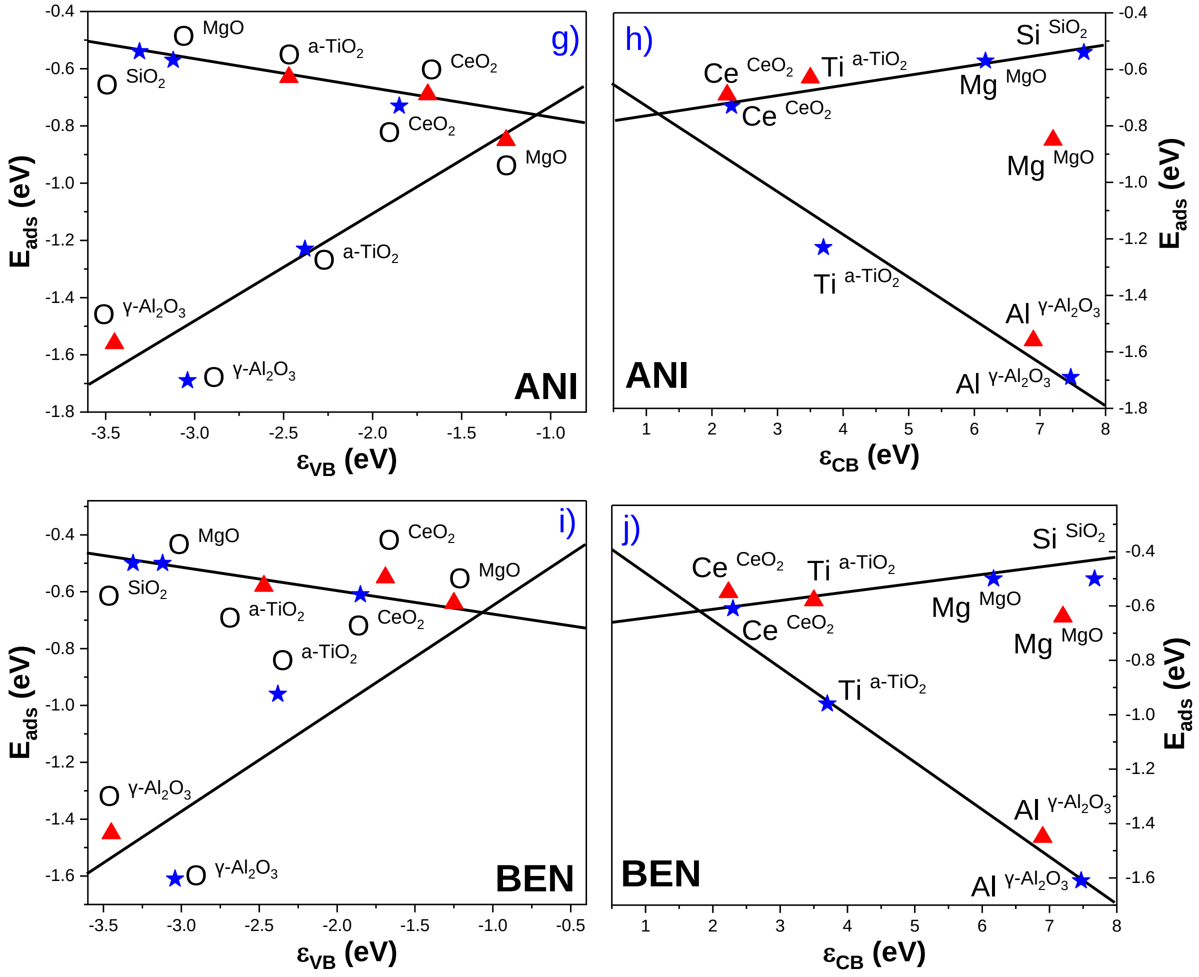

Figure S15. *Adsorption energies for the model compounds versus band centres (ε_{VB} and ε_{CB}) for the five oxide surfaces (clean and hydroxylated). Colour code: Red triangle (▲) and blue star (★) represents the clean and the hydroxylated oxide surfaces respectively.*

10. References

[1] Ziambaras, E.S., Elsebeth, *Physical Review B*. **2003**, *68*, 064112.

[2] Perdew, J.P.B., Kieron//Ernzerhof, Matthias, *Physical review letters*. **1996**, *77*, 3865.

[3] Aaron Deskins, N., D. Mei, and M. Dupuis, *Surface Science*. **2009**, *603*, 2793-2807.

[4] Lu, Y.-H. and H.-T. Chen, *Physical Chemistry Chemical Physics*. **2015**, *17*, 6834-6843.

[5] Digne, M., P. Sautet, P. Raybaud, P. Euzen, and H. Toulhoat, *Journal of Catalysis*. **2004**, *226*, 54-68.

[6] Gutiérrez, G., A. Taga, and B. Johansson, *Physical Review B*. **2001**, *65*, 012101.

[7] Quaino, P., O. Syzgantseva, L. Siffert, F. Tielens, C. Minot, and M. Calatayud, *Chemical Physics Letters*. **2012**, *519-520*, 69-72.

[8] Arasa, C., P. Gamallo, and R. Sayós, *The Journal of Physical Chemistry B*. **2005**, *109*, 14954-14964.

[9] Bredow, T. and A.R. Gerson, *Physical Review B*. **2000**, *61*, 5194.

[10] Del Vitto, A., G. Pacchioni, F. Delbecq, and P. Sautet, *The Journal of Physical Chemistry B*. **2005**, *109*, 8040-8048.

[11] Finazzi, E., C. Di Valentin, G. Pacchioni, and A. Selloni, *The Journal of chemical physics*. **2008**, *129*, 154113.

[12] Matz, O. and M. Calatayud, *The Journal of Physical Chemistry C*. **2017**, *121*, 13135-13143.

[13] Engel, J., S. Francis, and A. Roldan, *Physical Chemistry Chemical Physics*. **2019**.
